# Targeting chemokine-driven metastasis in non-small cell lung cancer: Development and evaluation of chemokine nanosponges for therapy

**DOI:** 10.1016/j.mtbio.2025.102511

**Published:** 2025-11-04

**Authors:** Libao Liu, Yonghui Wu, Weibin Wu, Zining Liu, Bolin Chen, Guanghong Wu, Zhe Ji, Jiannan Xu, Shuai Huang, Kai Zhang

**Affiliations:** a Department of Thoracic Surgery, The Third Affiliated Hospital of Sun Yat-sen University, Guangzhou, 510630, Guangdong Province, China; b Southern Medical University, Guangzhou, 510515, Guangdong Province, China; c Center for Information Technology and Statistics, The First Affiliated Hospital of Sun Yat-sen University, Guangzhou, Guangdong, 510080, China

**Keywords:** Non-small cell lung cancer (NSCLC), Chemokines and metastasis, Single-cell RNA sequencing (scRNA-seq), Tumor-associated macrophages (TAMs), Chemokine nanosponges

## Abstract

Non-small cell lung cancer (NSCLC) is the most common form of lung cancer, with a poor prognosis and high metastasis rate. Metastasis involves complex mechanisms, including chemokine secretion by tumor-associated macrophages (TAMs). Using single-cell RNA sequencing (scRNA-seq), we identified enhanced chemokine secretion by M2-type TAMs in metastatic lesions, with CCL20 emerging as a key target. We designed a CCL20-adsorbing nanosponge by engineering macrophages with high CCR6 expression and extracting their membranes. This nanosponge combines targeting ability and chemokine adsorption capacity, enabling precise treatment of high-CCL20 tumors. Additionally, we encapsulated the Toll-like receptor 7/8 agonist R848 within the CCR6-modified macrophage membrane (CCR6-MM) to polarize M2-type TAMs to the M1 phenotype, reducing CCL20 secretion and transforming the immunosuppressive tumor microenvironment. In vitro and in vivo experiments validated the therapeutic potential of the CCR6-MM and R848 combination, demonstrating biocompatibility, macrophage polarization efficacy, and dual inhibitory effects on tumor growth and metastasis. Our findings highlight the potential of chemokine nanosponges as a novel therapeutic strategy for NSCLC metastasis.

## Highlights

• CCL20 identified as a key chemokine in NSCLC metastasis, promoting an immunosuppressive tumor microenvironment.
• CCR6-MM@PS/R848 nanosponge engineered to target and adsorb CCL20, enhancing antitumor immunity.
• CCR6-MM@PS/R848 effectively inhibits tumor growth and metastasis, demonstrating strong therapeutic potential in NSCLC.

## Graphical abstract

**Figure:**
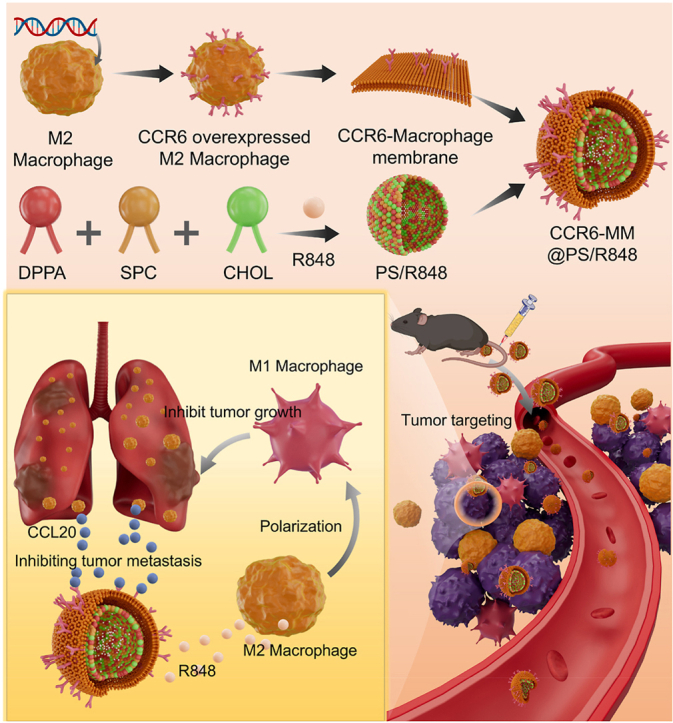

## 1 Introduction

Lung cancer is a prevalent malignancy, and non-small cell lung cancer (NSCLC) constitutes the majority of lung cancer cases, making up around 85 % of the total occurrences [1]. Even though there have been remarkable improvements in the diagnosis and treatment of NSCLC, the overall prognosis for patients is still unfavorable. The 5-year survival rate for NSCLC patients is relatively low, ranging from 15 % to 30 % [2,3]. Metastasis plays a crucial role in the failure of treatment and high mortality rate in NSCLC. It is worth noting that approximately one-third of NSCLC patients are diagnosed with metastases at the initial stage [4]. The metastatic process of NSCLC is intricate, encompassing various biological mechanisms. These mechanisms include the invasion of tumor cells, the formation of new blood vessels (angiogenesis), and the evasion of the immune system[[5], [6], [7]]. Recent studies have identified various molecular pathways, anatomical features, and genetic traits that contribute to the metastatic potential of NSCLC. These include hypoxia-induced epithelial-mesenchymal transition (EMT) [8], immune evasion through the PD-1/PD-L1 pathway [9], and the role of specific gene mutations such as EGFR [10] and KRAS [11]. Among the numerous mechanisms underlying NSCLC metastasis, the secretion of chemokines is a widely recognized factor [12].

Chemokines are small signaling proteins that play a crucial role in regulating immune cell recruitment and tumor progression[[13], [14], [15], [16]]. For instance, the chemokine CCL2 has been shown to promote tumor growth and metastasis by recruiting macrophages and other immune cells to the tumor microenvironment (TME) [17]. Tumor-associated macrophages (TAMs) are key regulators of the tumor microenvironment. They polarize into M1 macrophages (iNOS^+^CD80^+^) that produce IL-12 and TNF-α to suppress tumor growth, and M2 macrophages (Arg-1^+^CD206^+^) that secrete IL-10, CCL20 and other cytokines to drive tumor progression and metastasis [18,19]. Despite these findings, significant gaps remain in our understanding of the relationship between chemokines and tumor metastasis. Tumor heterogeneity poses a major challenge, making it difficult to establish a clear correlation between chemokine expression and metastatic potential. Additionally, the sources and regulatory mechanisms of chemokine production in the TME are not fully elucidated [20].

Single-cell RNA sequencing (scRNA-seq) has recently gained prominence in molecular biology, providing detailed information on cellular heterogeneity and gene expression at the single-cell level [[21], [22], [23]]. Traditional bulk RNA sequencing offers an average gene expression profile for all cells in a sample, whereas scRNA-seq enables the analysis of gene expression in individual cells, uncovering the true complexity and diversity of cellular populations [[24], [25], [26]]. This technology has several key advantages, such as resolving cellular heterogeneity, detecting rare cell types, and generating cell type-specific gene expression profiles. In the context of NSCLC, scRNA-seq has been crucial in understanding the complex cellular interactions within the TME. In our research, we utilized scRNA-seq combined with bioinformatics analysis to explore the relationship between NSCLC metastasis and chemokine expression levels. Our analysis demonstrated that M2-type TAMs in metastatic lesions have enhanced chemokine secretion functions, with CCL20 identified as a key target.

Several therapeutic strategies targeting chemokines have been explored, including the use of monoclonal antibodies, small molecule inhibitors, and engineered cytokines [27]. However, these approaches face significant challenges due to the lack of tumor targeting and poor biocompatibility [13]. For example, clinical trials targeting the CCL2-CCR2 axis have shown limited efficacy, with most drugs being discontinued due to lack of response [13]. Thanks to advancements in nano-biotechnology, cell membrane engineering has emerged as a novel treatment method [28]. Cell membranes, particularly those derived from macrophages, possess inherent targeting capabilities and biocompatibility, making them ideal for delivering therapeutic agents directly to the tumor site [29,30]. Macrophage membranes can be engineered to express specific receptors or ligands, enhancing their ability to target tumors and modulate the TME. For example, macrophage membranes expressing chemokine receptors such as CCR2 has shown potential in delivering drugs to tumors and improving therapeutic efficacy [31]. Additionally, cell membrane-coated nanoparticles (CNPs) have been developed to enhance drug delivery[[32], [33], [34]]. These CNPs combine the targeting ability of macrophage membranes with the drug-carrying capacity of synthetic nanoparticles, providing a multifunctional platform for cancer therapy.

**Scheme 1:**
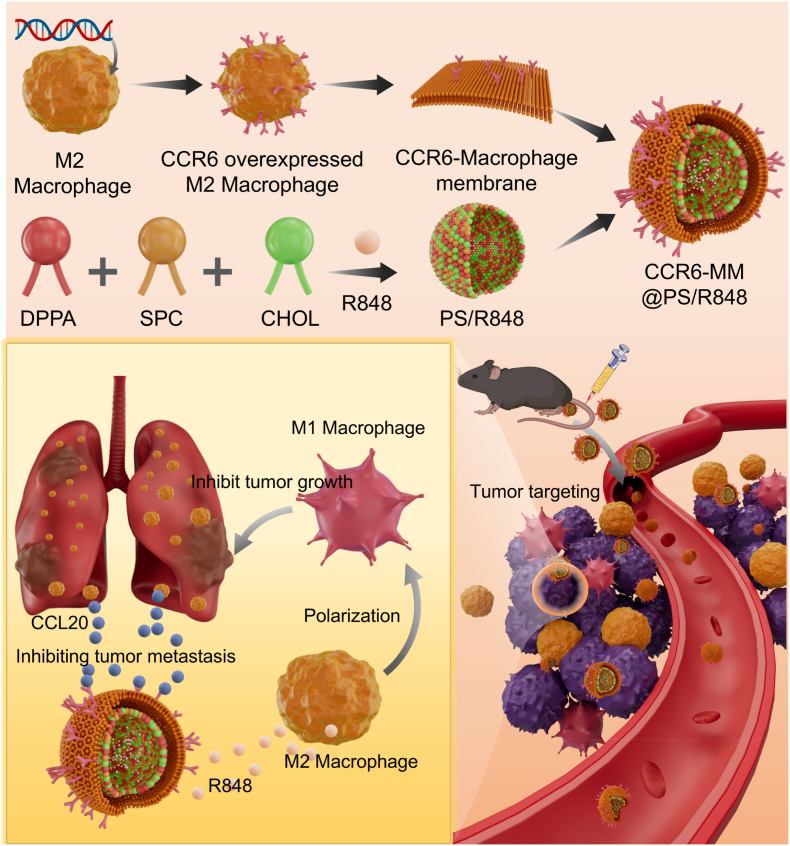
Schematic illustration of the preparation and mechanism of CCR6-MM@PS/R848 for NSCLC treatment.

Based on the aforementioned background, we designed a nanosponge capable of adsorbing chemokine CCL20 (Scheme 1). Leveraging the ligand-receptor interaction between CCL20 and CCR6, we engineered macrophages with high CCR6 expression and extracted their membranes to construct the nanosponge. This nanosponge combines the targeting ability of macrophage membranes with the chemokine adsorption capacity, enabling precise treatment of tumors with high CCL20 levels. Additionally, we utilized the macrophage membrane as a carrier for nanodrugs, enhancing drug targeting and biocompatibility. We further encapsulated the Toll-like receptor 7/8 agonist R848 within the CCR6-modified macrophage membrane (CCR6-MM) to polarize M2-type macrophages to the M1 phenotype [35,36], thereby reducing CCL20 secretion and transforming the immunosuppressive TME into an immunogenic one. Our study validated the therapeutic potential of the CCR6-MM and R848 combination (CCR6-MM@PS/R848) through in vitro and in vivo experiments, demonstrating its biocompatibility, macrophage polarization efficacy, and dual inhibitory effects on tumor growth and metastasis.

## 2 Materials and methods

### 2.1 Acquisition and analysis of scRNA-seq data

In the present study, we retrieved the GSE131907 dataset from the Gene Expression Omnibus (GEO), which contains single-cell RNA sequencing (scRNA-seq) data from lung tissue samples of patients with primary NSCLC and their corresponding metastatic lesions. The "Seurat" R package was employed to process the data and generate a Seurat object. For quality control, cells with fewer than 300 or more than 6000 detected features, total UMI counts below 500 or above 7000, and mitochondrial gene expression exceeding 15 % (pctMT >15 %) were excluded.

Next, the FindVariableFeatures function was used to identify the top 2000 highly variable genes for further analysis. Data normalization was performed, followed by principal component analysis (PCA) using the ScaleData and RunPCA functions. To correct for batch effects, the Harmony algorithm was applied for data integration. A k-nearest neighbors (KNN) graph was constructed based on the first 35 principal components using the FindNeighbors function. Cell clustering was then conducted with the FindClusters function at a resolution of 0.1. Finally, Uniform Manifold Approximation and Projection (UMAP) was utilized for dimensionality reduction and visualization. Major cell types and subtypes were annotated and visualized using canonical marker genes to assess cell-type-specific expression patterns.

### 2.2 Differential analysis, functional enrichment analysis, and survival analysis

To identify differentially expressed genes (DEGs), the FindMarkers function from the Seurat package was employed to compare gene expression levels between the tumor and metastasis groups. The selection of DEGs was based on the Wilcoxon rank-sum test, with criteria set at an absolute log2 fold change (log2FC) exceeding 0.5 and a P-value below 0.05. Subsequently, the upregulated genes were analyzed for functional annotation and pathway enrichment using the Gene Ontology (GO) and Kyoto Encyclopedia of Genes and Genomes (KEGG) databases. Pathways or functions with a P-value of 0.05 or lower were considered significantly enriched. Survival analysis was performed using the GEPIA online platform (http://gepia.cancer-pku.cn/index.html), with the 50 % median cutoff point used to evaluate overall survival (OS) and disease-free survival (DFS), and the corresponding survival curves were generated.

### 2.3 Construction of CCR6 overexpression in RAW264.7 cells

To establish CCR6-overexpressing RAW264.7 cells, the CCR6 gene was inserted into a lentiviral vector (pIRES2-EGFP, Vigene Biosciences, China). Lentivirus was generated by cotransfecting 293T cells with the CCR6 vector and packaging plasmids (Vigene Biosciences) using Lipofectamine 3000 (Thermo Fisher Scientific). The viral supernatant was collected, filtered, and concentrated via ultracentrifugation. RAW264.7 cells were infected with the lentivirus at a multiplicity of infection (MOI) of 50 in the presence of 8 μg/mL polybrene (Solarbio, China). Stable transduced cells were selected using 1 μg/mL puromycin (Solarbio, China) for 2 weeks. The overexpression of CCR6 was verified by quantitative real-time polymerase chain reaction (qRT-PCR), immunofluorescence, and flow cytometry.

### 2.4 Preparation and characterization of CCR6-MM@PS/R848

The PS/R848 liposome was synthesized via the thin-film hydration method. Specifically, 985.4 mg of lecithin, 227.4 mg of 1,2-dipalmitoyl-d62-sn-glycero-3-phosphoethanolamine, and 154.6 mg of cholesterol were dissolved in a mixture of 40 mL chloroform and 8 mL ethanol. Subsequently, 1 mg/mL of resiquimod (R848) was incorporated into the mixture. This solution was magnetically stirred in a water bath at 37 °C for 60 min until the drug was fully dissolved. The mixture was then transferred to a rotary evaporator and maintained at 52 °C for 30 min to form a yellow film on the glass wall. Next, 40 mL of PBS (pH 7.4, 10 × 10−3 M) was added to the mixture, followed by an additional 30 min of stirring. Finally, the resultant solution was transferred to a 100 mL beaker and subjected to ultrasonication at 200 W and 25 °C for 5 min. The synthesis of PS liposomes followed the same procedure, except that R848 was omitted.

Cell membrane hybrid liposomes (CCR6-MM@PS/R848) were fabricated using an extrusion method with an extruder from Avanti Polar Lipids, Inc. (USA). Specifically, PS/R848 liposomes were mixed with cell membrane proteins at a weight ratio of 1:1. This mixture was sonicated in an ice-water bath for 5 min. After sonication, the mixture was extruded ten times through polycarbonate porous membrane filters with pore sizes of 800 nm, 400 nm, and 200 nm, respectively, at 37 °C. Subsequently, free drugs were removed from the mixture using ultrafiltration tubes (Millipore, molecular weight cutoff of 3000 Da). To visualize the liposome and CCR6-MM membranes, the lipophilic dyes DiD (excitation/emission maxima at 644 nm/665 nm) and DiO (excitation/emission maxima at 484 nm/501 nm) were employed. The nanovesicles with dye-labeled membranes were examined using confocal laser scanning microscopy to assess their structural integrity.

The hydrodynamic diameter and zeta potential of PS, PS/R848, and CCR6-MM@PS/R848 were measured by dynamic light scattering (DLS) using a Zetasizer 3000 (Malvern, USA). Each measurement was performed three times to obtain the average value and determine the polydispersity index (PDI). The morphology of the liposomes was examined using a transmission electron microscope (TEM, HT-7700, Hitachi, Japan) at 200 kV. A drop of the liposome solution was placed on a copper grid coated with amorphous carbon and stained with a 2 % phosphotungstic acid solution.

### 2.5 Drug loading capacity and drug release manner of hybrid liposomes

Following the preparation of the hybrid liposomes, ultrafiltration was utilized to remove unencapsulated free drugs. The encapsulated drugs were subsequently extracted using ethanol and quantified by UV spectrophotometry (UV, UH5300, Japan). Standard curves for the drugs were concurrently established. The encapsulation efficiency was evaluated as the ratio of the encapsulated drug to the total drug dose, expressed as a percentage. The loading efficiency was determined by calculating the proportion of the encapsulated drug relative to the total mass of the liposomes, also presented as a percentage.

To investigate the drug release profile from the liposomes, an experiment was performed. Specifically, 1 mL of CCR6-MM@PS-R848 was placed into a dialysis tube with a molecular weight cutoff (MWCO) of 3500 Da. This tube was then immersed in 19 mL of phosphate-buffered saline (PBS) at pH 7.4 and a concentration of 10 mM. The setup was maintained at 37 °C and subjected to continuous orbital shaking at a speed of 200 rpm. At predetermined time intervals (0, 12, 24, 48, and 72 h), 0.5 mL of the PBS was collected and replaced with an equal volume of fresh PBS. The amount of drug released was quantified using UV spectroscopy.

### 2.6 Coomassie Brilliant Blue staining

For the visualization of proteins in polyacrylamide gels, Coomassie Brilliant Blue staining was utilized. Following electrophoresis, the gels were carefully extracted from the glass plates and briefly rinsed with distilled water to eliminate any residual SDS. Subsequently, the gels were fixed in a solution comprising 40 % methanol and 10 % acetic acid for 30 min to stabilize the proteins and enhance band resolution. After fixation, the gels were stained with Coomassie Brilliant Blue R-250 (Sigma-Aldrich, USA). The gels were immersed in the staining solution for 1 h with gentle agitation to ensure uniform staining. Following staining, the gels were destained in a solution containing 40 % methanol and 10 % acetic acid to remove excess dye. The destaining process involved multiple changes of the destaining solution until the background was clear. Finally, the gels were imaged using a gel documentation system (Bio-Rad Laboratories, Hercules, USA) to visualize the protein bands.

### 2.7 In vivo imaging of material distribution in excised organs and tumors

To explore the distribution of R848 in excised organs and tumors, an in vivo imaging system (IVIS) was utilized. R848 was labeled with Cy5.5 dye (Thermo Fisher Scientific, USA) to facilitate the visualization of its biodistribution. The conjugation of R848 to the fluorescent dye was performed following standard protocols. Specifically, R848 was dissolved in a suitable buffer, and the fluorescent dye was added to promote efficient conjugation. The reaction mixture was incubated at room temperature for 2 h, followed by purification using a desalting column (Sephadex G-25, GE Healthcare, USA) to eliminate unbound dye. Mice harboring subcutaneous tumors were intravenously injected with the PS/R848-Cy5.5 and CCR6-MM@PS/R848-Cy5.5 conjugates. At 48 h post-injection, the mice were euthanized, and the excised organs (liver and kidneys) and tumors were collected. The livers, kidneys, and tumors were placed in a black imaging cassette to reduce background fluorescence. Fluorescence images of the excised tissues were captured using the IVIS system.

### 2.8 Cell culture

The murine macrophage cell line RAW264.7, the murine fibroblast cell line L929, and the Lewis lung carcinoma cell line LLC were obtained from the American Type Culture Collection (ATCC, USA). These cell lines were cultured in Dulbecco's Modified Eagle Medium (DMEM) from Gibco (Thermo Fisher Scientific, USA). The culture media were supplemented with 10 % fetal bovine serum (FBS, Gibco), 100 units/mL of penicillin, and 100 μg/mL of streptomycin (Sigma-Aldrich, USA). The cells were maintained at 37 °C in an atmosphere containing 5 % CO_2_, with the culture medium being changed every 2–3 days. When the cells reached 80 % confluence, they were passaged using a 0.25 % trypsin-EDTA solution (Sigma-Aldrich).

### 2.9 Live/dead staining assay

L929 cells were seeded at a density of 5 × 10^4^ cells per well in a 24-well plate and permitted to adhere and proliferate for 24 h. Following this initial incubation period, the cells were exposed to various treatments for an additional 24 h, including control, CCR6-MM, R848, and CCR6-MM@PS/R848. After treatment, the cells were washed twice with PBS to remove any residual treatment agents. The live-dead staining kit (Invitrogen, USA) was utilized according to the manufacturer's instructions. Specifically, the cells were incubated with a staining solution containing Calcein-AM (a green fluorescent dye that labels live cells) and PI (a red fluorescent dye that labels dead cells) for 30 min at 37 °C in the dark. Subsequently, the cells were rinsed with PBS and examined under a fluorescence microscope (Olympus, Japan). Cell viability was evaluated by counting the number of live (green) and dead (red) cells in five random fields per well.

### 2.10 CCK-8 assay

L929 cells were seeded at a density of 1 × 10^4^ cells per well in a 96-well plate and permitted to adhere and proliferate for 24 h. Following the initial incubation, the cells were subjected to various treatments for an additional 24 h, including control, CCR6-MM, R848, and CCR6-MM@PS/R848. After treatment, the cells were washed twice with PBS to eliminate any residual treatment agents. The CCK-8 assay was performed according to the manufacturer's protocol (C0039, Beyotime Biotechnology). Specifically, 10 μL of CCK-8 solution was added to each well, and the cells were incubated for 2 h at 37 °C in a humidified atmosphere containing 5 % CO_2_. The absorbance was measured at 450 nm using a microplate reader (Thermo Fisher Scientific, USA). Cell viability was expressed as a percentage relative to the control group.

### 2.11 In vitro hemolysis assay

Fresh red blood cells (RBCs) from mice were collected and washed three times with PBS to remove any residual plasma proteins. The RBCs were then resuspended in PBS to obtain a 5 % hematocrit level. The experiment was set up with three groups: control (PBS only), CCR6-MM@PS/R848, and Triton-X (positive control), with each group prepared in triplicate. A 100 μL volume of the RBC suspension was mixed with 100 μL of each treatment in a 96-well plate. For the positive control, Triton-X was used at a concentration of 0.1 %. The plate was incubated at 37 °C for 1 h. After incubation, the samples were centrifuged at 3000 rpm for 10 min to pellet the RBCs. The supernatant was carefully transferred to a new plate, and the absorbance was measured at 540 nm using a microplate reader (Thermo Fisher Scientific, USA) to quantify the released hemoglobin.

### 2.12 Hematological and biochemical analysis

Blood samples were obtained from the orbital sinus on day 7 following treatment. These samples were analyzed for various hematological and biochemical parameters, including white blood cell count (WBC), red blood cell count (RBC), platelet count (PLT), hemoglobin (HGB), hematocrit (HCT), blood urea nitrogen (BUN), creatinine (CREA), granulocytes (GRAN), aspartate aminotransferase (AST), and alanine aminotransferase (ALT). The analyses were conducted using an automated hematology analyzer (Sysmex, Japan) and a biochemical analyzer (Hitachi, Japan).

### 2.13 Organ biocompatibility experiment

On day 7 post-treatment, the mice were humanely sacrificed, and major organs such as the heart, liver, spleen, lung, and kidney were harvested for histopathological examination. These organs were fixed in 10 % neutral buffered formalin, embedded in paraffin, and sectioned at a thickness of 5 μm. The sections were then stained with hematoxylin and eosin (H&E). A light microscope (Olympus, Japan) was employed to observe the sections for any signs of tissue damage or inflammation.

### 2.14 Immunofluorescence assay

Raw264.7 cells were seeded at a density of 1 × 10^5^ cells per well in a 24-well plate and permitted to adhere and proliferate for 24 h. Following the initial incubation period, the cells were subjected to various treatments for an additional 24 h, including control, CCR6-MM, R848, and CCR6-MM@PS/R848. After treatment, the cells were washed twice with PBS and fixed with 4 % paraformaldehyde for 15 min at room temperature. Subsequently, the cells were permeabilized with 0.1 % Triton X-100 in PBS for 10 min and blocked with 5 % bovine serum albumin (BSA) in PBS for 1 h at room temperature. The cells were then incubated overnight at 4 °C with primary antibodies against CCR6 (Abcam, ab134074, 1:100), iNOS (Abcam, ab15323, 1:100), and Arg-1 (Abcam, ab198897, 1:100) in 5 % BSA in PBS. After washing three times with PBS, the cells were incubated with Alexa Fluor 488-conjugated anti-rabbit secondary antibody (Abcam, ab150077, 1:500) for 1 h at room temperature in the dark. The nuclei were stained with Hoechst 33342 (10 μg/mL) for 10 min at room temperature in the dark. The cells were observed under a fluorescence microscope (Olympus, Japan) and images were captured at 20 × magnification. The fluorescence intensity of the target proteins was quantified using ImageJ software.

### 2.15 q-PCR analysis

Total RNA was extracted using the TRIzol reagent (Invitrogen, USA) according to the manufacturer's instructions. The concentration and purity of the RNA were measured using a NanoDrop spectrophotometer (Thermo Fisher Scientific, USA). RNA integrity was confirmed by agarose gel electrophoresis. Next, 1 μg of total RNA was reverse-transcribed into cDNA using the PrimeScript RT Reagent Kit (Takara, Japan) following the manufacturer's protocol. Quantitative PCR (q-PCR) was performed using the SYBR Green Master Mix (Applied Biosystems, USA) on a StepOnePlus Real-Time PCR System (Applied Biosystems). The primers used are listed as follows:

**Table:** 

Target	Forward	Reverse
iNOS	GTTCTCAGCCCAACAATACAAGA	GTGGACGGGTCGATGTCAC
Arg-1	CTCCAAGCCAAAGTCCTTAGAG	GGAGCTGTCATTAGGGACATCA
CCR6	CACACCTGTGAGAGGAAGCA	AGCAGAGGTGAAGCAATAATGT
GAPDH	AGACAGCCGCATCTTCTTGT	CTTGCCGTGGGTAGAGTCAT

The PCR thermal cycling conditions were as follows: an initial denaturation at 95 °C for 10 min, followed by 40 cycles of 95 °C for 15 s and 60 °C for 1 min. The relative expression levels of the target genes were calculated using the 2^−ΔΔCt^ method, with GAPDH used as the internal control.

### 2.16 Flow cytometry analysis

Raw264.7 cells were seeded at a density of 1 × 10^6^ cells per well in a 6-well plate and permitted to adhere and proliferate for 24 h. Following the initial incubation period, the cells were subjected to various treatments for an additional 24 h, including control, CCR6-MM, R848, and CCR6-MM@PS/R848. After treatment, the cells were harvested, washed twice with PBS, and resuspended in PBS at a concentration of 1 × 10^6^ cells/mL. The cells were then incubated with primary antibodies against F4/80 (123120, BioLegend), CD86 (159216, BioLegend), and CD206 (141726, BioLegend) in PBS containing 1 % BSA for 30 min at 4 °C in the dark. After washing with PBS, the stained cells were analyzed using a flow cytometer (BD FACSCalibur, BD Biosciences), and data were acquired and analyzed using FlowJo software (Tree Star, Inc.) to determine the percentage of positive cells for each marker.

### 2.17 WB assay

Raw264.7 cells were seeded at a density of 1 × 10^6^ cells per well in a 6-well plate and permitted to adhere and proliferate for 24 h. Following the initial incubation period, the cells were subjected to various treatments for an additional 24 h, including control, CCR6-MM, R848, and CCR6-MM@PS/R848. The cells were lysed using RIPA buffer supplemented with protease inhibitors (PMSF) to prevent protein degradation. The protein concentration was measured using the BCA Protein Assay Kit (Thermo Fisher Scientific). Protein samples were denatured by boiling in 5 × loading buffer for 10 min, separated by SDS-PAGE using a 10 % polyacrylamide gel, and transferred to a 0.45 μm PVDF membrane (Millipore) using a wet transfer system at 200 mA for 2 h. The membrane was blocked with 5 % skim milk in TBST (TBS with 0.1 % Tween-20) for 1 h at room temperature, and incubated overnight at 4 °C with primary antibodies against iNOS (Abcam, ab178945, 1:1000) and Arg-1 (Abcam, ab226165, 1:1000) in 5 % skim milk in TBST. After washing with TBST, the membrane was incubated with HRP-conjugated anti-rabbit secondary antibody (Abcam, ab97051, 1:5000) for 1 h at room temperature. The proteins were detected using an ECL detection kit (Thermo Fisher Scientific) and captured using a BLT Imaging System. The intensity of the bands was quantified using ImageJ software.

### 2.18 ELISA assay

Raw264.7 cells were seeded at a density of 1 × 10^6^ cells per well in a 6-well plate and permitted to adhere and proliferate for 24 h. Following the initial incubation period, the cells were subjected to various treatments for an additional 24 h, including control, CCR6-MM, R848, and CCR6-MM@PS/R848. The supernatants were collected and centrifuged at 1000 rpm for 10 min to remove any cellular debris. The levels of TNF-α and IL-10 in the supernatants were measured using ELISA kits (Abcam) according to the manufacturer's instructions. Specifically, 96-well plates were coated with capture antibodies specific to TNF-α (Abcam, ab208348) and IL-10 (Abcam, ab210563) overnight at 4 °C. The plates were then washed with PBST and blocked with 1 % BSA in PBST for 1 h at room temperature. Supernatant samples and standard solutions were added to the wells and incubated for 2 h at room temperature. Detection antibodies specific to TNF-α (Abcam, ab208348) and IL-10 (Abcam, ab210563) were added and incubated for 1 h at room temperature. After washing with PBST, HRP-conjugated secondary antibodies were added and incubated for 30 min at room temperature. The substrate solution (TMB) was added to each well and incubated for 15 min in the dark. The reaction was terminated by adding 50 μL of 2 M H2SO4 to each well. The absorbance was measured at 450 nm using a microplate reader (Thermo Fisher Scientific). The concentration of TNF-α and IL-10 in the samples was determined by comparing the absorbance values to a standard curve generated from the standard solutions.

### 2.19 Subcutaneous lung cancer animal model and treatment

Healthy female C57BL/6 mice, aged 6–8 weeks, were selected for this study. The animal experimental protocols were approved by the Animal Ethics Committee of Sun Yat-sen University (L102012024045K, Guangzhou, China) and were strictly conducted in accordance with the Regulations for the Administration of Affairs Concerning Experimental Animals of China. To establish a subcutaneous Lewis lung carcinoma tumor model in these mice, the following procedures were performed. LLC cells were cultured in RPMI 1640 medium, supplemented with 10 % fetal bovine serum, penicillin, and streptomycin, until they reached the logarithmic growth phase. The cells were then collected, and their concentration was adjusted to 5 × 10^6^ cells/mL using sterile PBS. Under sterile conditions, 100 μL of the cell suspension was injected subcutaneously into the right flank of each mouse. Tumor growth was monitored by measuring the tumor dimensions with calipers every 3 days, and the tumor volume was calculated using the formula: V = 1/2 × length × width^2^. Once the tumor volume reached approximately 100–150 mm^3^, the mice were randomly assigned to four treatment groups: control (saline), CCR6-MM, R848, and CCR6-MM@PS/R848. The treatments were administered intravenously twice per week for a total of four doses. Throughout the experiment, tumor volume and body weight were measured to assess treatment efficacy and potential toxicity. The experiment was terminated when the tumor volume reached approximately 1500 mm^3^ or if the mice showed signs of distress. At the end of the study, the tumors were excised, weighed, and subjected to further analysis.

### 2.20 Immunohistochemistry assay

The excised tumors were fixed in 4 % paraformaldehyde for 24 h at room temperature and subsequently processed for paraffin embedding. Sections with a thickness of 4 μm were cut and mounted on glass slides. These paraffin-embedded sections were deparaffinized, rehydrated, and subjected to antigen retrieval in citrate buffer (pH 6.0) using a microwave for 15 min. The sections were treated with 3 % hydrogen peroxide to quench endogenous peroxidase activity, blocked with 5 % BSA, and incubated overnight at 4 °C with primary antibodies against Ki67 (Abcam, ab15580, 1:200), CCL20 (Abcam, ab9829, 1:100), Arg-1 (Abcam, ab226165, 1:100), and iNOS (Abcam, ab178945, 1:100). After washing, the sections were incubated with HRP-conjugated secondary antibodies (Abcam, ab97051, 1:500) for 30 min at room temperature, developed using a DAB substrate kit (Abcam, ab64238), counterstained with hematoxylin, dehydrated, and mounted. The stained sections were imaged using a light microscope (Olympus, Japan) at 20 × magnification. The percentage of positive cells was quantified in five randomly selected fields per section.

### 2.21 Transwell assay

For the migration assay, 24-well Transwell inserts with 8 μm pore size polycarbonate membranes (Corning) were used. The lower chamber was filled with the supernatant from Raw264.7 cells treated under various conditions. The upper chamber was seeded with 100 μL of LLC cell suspensions (1 × 10^5^ cells). After incubation for 24 h at 37 °C in a humidified atmosphere with 5 % CO_2_, non-migrated cells on the upper surface of the membrane were removed using a cotton swab. Migrated cells on the lower surface were fixed with 4 % paraformaldehyde for 15 min and stained with 0.1 % crystal violet for 10 min. The number of migrated cells was quantified by counting five random fields per well under a light microscope at 20 × magnification.

For invasion assays, the upper chamber was coated with Matrigel, and cells were allowed to invade for 48 h. Subsequently, invasive cells were processed and quantified in a similar manner to the migration assays.

### 2.22 Wound healing assay

LLC cells were cultured in DMEM supplemented with 10 % FBS until they reached 80 % confluence in 6-well plates. The cells were then serum-starved for 24 h to synchronize their growth. A uniform scratch was created in the monolayer using a sterile 200 μL pipette tip, and the cells were gently washed with PBS to remove any detached cells. The scratch was imaged at 0 h using a light microscope (Olympus, Japan). The culture medium was replaced with supernatants from Raw264.7 cells treated with different formulations: control (saline), PS/R848, MM@PS/R848, and CCR6-MM@PS/R848. The plates were incubated at 37 °C in a humidified atmosphere with 5 % CO_2_, and images of the scratch were captured at 24 h post-treatment using the same microscope settings. The migration of LLC cells into the scratch area was quantified by measuring the scratch width at multiple points using ImageJ software, and the percentage of scratch closure was calculated.

### 2.23 Lung metastasis animal model

To create a lung metastasis model using LLC cells and assess the impact of various treatments, a tail vein injection approach was utilized in female C57BL/6 mice. LLC cells were cultured in DMEM, supplemented with 10 % FBS, penicillin, and streptomycin, until they reached the logarithmic growth phase. The cells were then collected and adjusted to a concentration of 1 × 10^6^ cells/mL using sterile PBS. The mice were anesthetized, and their tails were warmed to dilate the veins. A 27-gauge needle was used to inject 100 μL of the cell suspension (containing 1 × 10^5^ cells) into the tail vein of each mouse. Following injection, the mice were divided into four treatment groups: control (saline), PS/R848, MM@PS/R848, and CCR6-MM@PS/R848. The treatments were administered intravenously twice per week for a total of four doses. At the conclusion of the study, the lungs were excised, fixed in 4 % paraformaldehyde, and subjected to histological analysis to confirm the presence of metastatic lesions.

### 2.24 Statistical analysis

Each experiment was independently repeated at least three times, with results presented as the mean ± standard deviation (SD) unless otherwise specified. For comparisons between two unpaired groups, an unpaired Student's t-test (two-tailed) was utilized. When comparing multiple groups, a one-way ANOVA was employed, followed by Tukey's post-hoc test for multiple comparisons. GraphPad Prism 9.0 was used to calculate P values, with significance levels indicated as *P < 0.05, **P < 0.01, and ***P < 0.001. The Kaplan-Meier method was used for survival analysis.

## 3 Results and discussion

### 3.1 TME remodeling in NSCLC metastasis

**Fig. 1:**
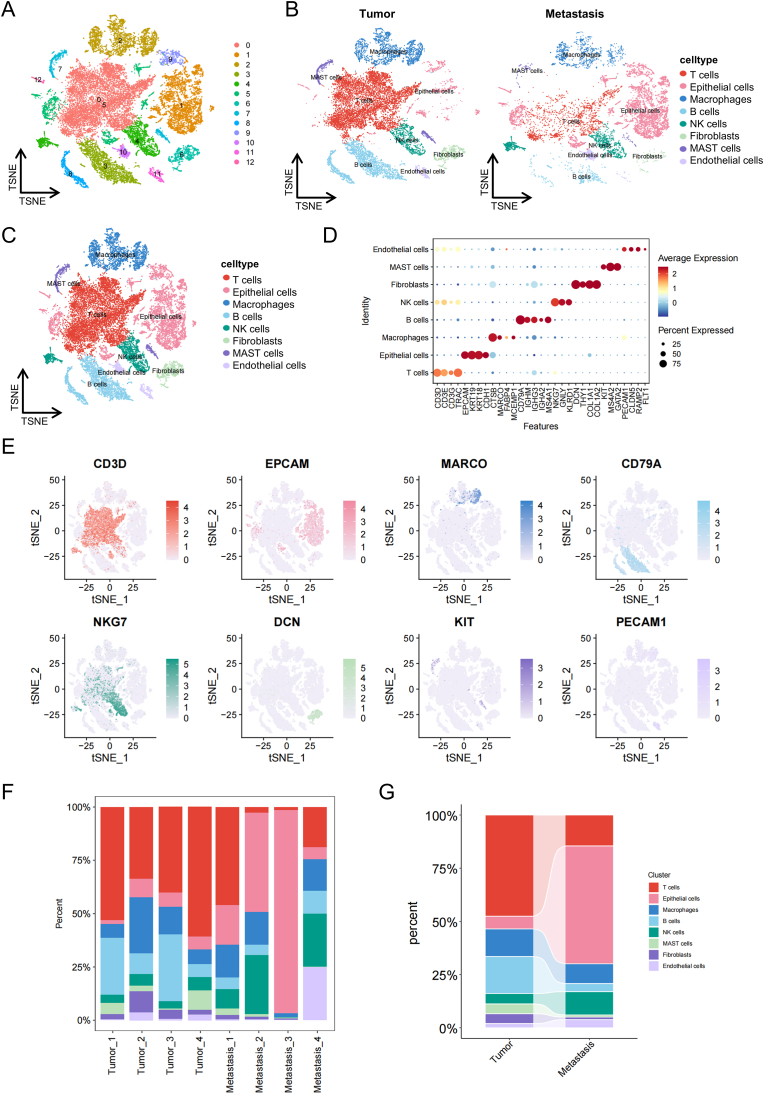
Comprehensive scRNA-seq analysis of tumor and metastatic lung tissue samples from NSCLC patients. (A) t-Distributed Stochastic Neighbor Embedding (t-SNE) plot depicting the distribution of single cells clustered into 13 distinct groups. (B) t-SNE plots of the tumor and metastatic groups following annotation, identifying eight major cell types. (C) Combined t-SNE plot showing all annotated cells. (D) Bubble plot highlighting the expression levels of marker genes across the identified cell types. (E) t-SNE plots illustrating the expression patterns of canonical marker genes for the eight major cell types. (F) Proportional representation of each cell type across all samples. (G) Comparison of cell type proportions between the tumor and metastatic groups.

To elucidate the changes in the TME during NSCLC metastasis, we analyzed the single-cell transcriptomic dataset GSE131907, which encompasses primary and metastatic lung tissues from NSCLC patients. Rigorous quality control measures were implemented to exclude low-quality data, ensuring the robustness of subsequent analyses. We identified 13 distinct cell clusters, each characterized and validated based on canonical markers and gene expression profiles (Fig. 1A). A total of 8 cell types were annotated, including T cells (marker genes: CD3D, TRAC), epithelial cells (marker genes: EPCAM, KRT19), macrophages (marker genes: CTSB, MARCO), B cells (marker genes: CD79A, IGHM), NK cells (marker genes: NKG7, GNLY), fibroblasts (marker genes: DCN, COL1A1), mast cells (marker genes: MS5A2, GATA2), and endothelial cells (marker genes: PECAM1, RAMP2) (Fig. 1B, C, 1D, and 1E). Fig. 1F and G illustrate the proportions of each cell type across different samples and groups. Notably, T cells, epithelial cells, macrophages, and B cells were found in higher proportions, while NK cells, fibroblasts, mast cells, and endothelial cells were relatively less abundant. Comparative analysis revealed significant alterations in cell proportions between metastatic and non-metastatic groups, with a notable decrease in T cells and B cells in the metastatic group. These findings suggest that lung cancer metastasis is closely associated with immune cell remodeling. The observed changes in immune cell proportions, particularly the reduction in T cells and B cells, highlight the dynamic nature of the TME during metastasis. This immune cell shift may contribute to immune evasion and tumor progression, underscoring the importance of targeting immune cell populations in therapeutic strategies.

### 3.2 Macrophage polarization and CCL20 expression in metastatic

**Fig. 2:**
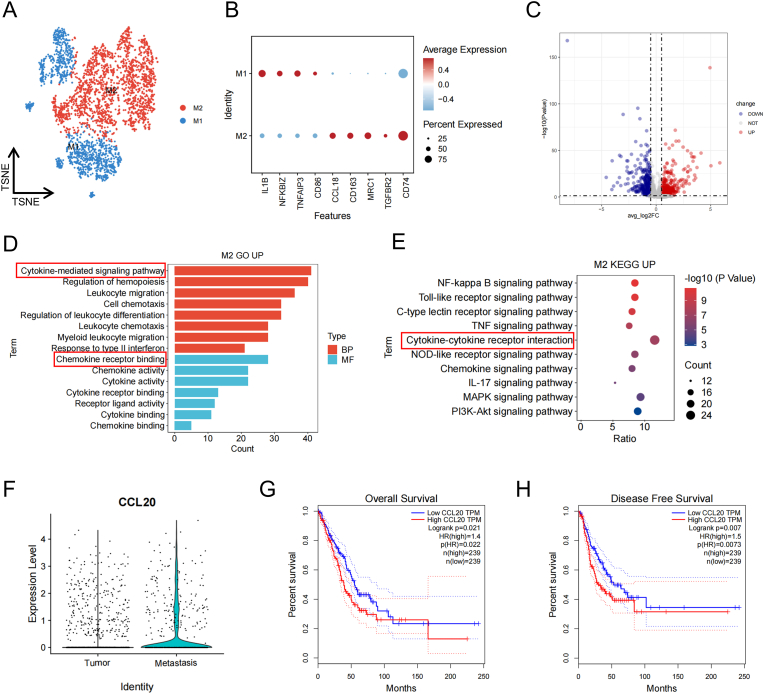
Subpopulation, functional, and prognostic analysis of macrophages. (A) t-SNE plot depicting the annotation results for macrophages. (B) Bubble plot showing the expression of classic marker genes for macrophages. (C) Volcano plot illustrating DEGs in M2 macrophages. (D) GO enrichment analysis of differentially expressed genes. (E) KEGG pathway enrichment analysis of differentially expressed genes. (F) Violin plot showing the mRNA expression levels of CCL20 in M2 macrophages between the tumor and metastatic groups. (G) Kaplan-Meier survival curve for CCL20 gene expression, showing OS. (H) Kaplan-Meier survival curve for CCL20 gene expression, showing DFS.

Macrophages are pivotal in the progression of metastatic NSCLC. TAMs are known to facilitate immune evasion and drive tumor metastasis through various mechanisms, including promoting tumor cell invasion and dissemination. We performed subgroup analysis of macrophages, identifying classical macrophages (M1) characterized by marker genes such as IL1B and TNFAIP3, and alternatively activated macrophages (M2) marked by CCL18 and CD163 (Fig. 2A and B). Differential analysis of M2 macrophages revealed upregulated genes, which were subjected to GO and KEGG enrichment analysis (Fig. 2C). GO analysis highlighted significant activation of pathways related to cytokine-mediated signaling and cytokine activity, indicating the broad regulatory role of cytokines in the metastatic TME (Fig. 2D). KEGG analysis further confirmed the upregulation of the cytokine-cytokine receptor pathway, reinforcing the critical role of cytokines in metastasis (Fig. 2E). To systematically evaluate which cytokine(s) within this pathway are functionally relevant, we quantified expression of all 11 gene annotated in the KEGG map and constructed corresponding Kaplan–Meier survival curves (Fig. S1). Among them, only CCL20 exhibited simultaneously (i) significantly higher expression in the metastatic cohort (log_2_FC = 1.42, adjusted p < 0.001) and (ii) a robust association with poor overall survival (HR = 1.40, p = 0.002) and disease-free survival (HR = 1.50, p = 0.001) (Fig. 2F–H). None of the other cytokines achieved statistical significance for both criteria (Fig. S1). These data identify CCL20 as the key cytokine candidate through which M2 macrophages potentiate NSCLC metastasis, and they provide a rational basis for therapeutically targeting the CCL20-CCR6 axis.

### 3.3 Characterization of CCR6-MM@PS/R848

**Fig. 3:**
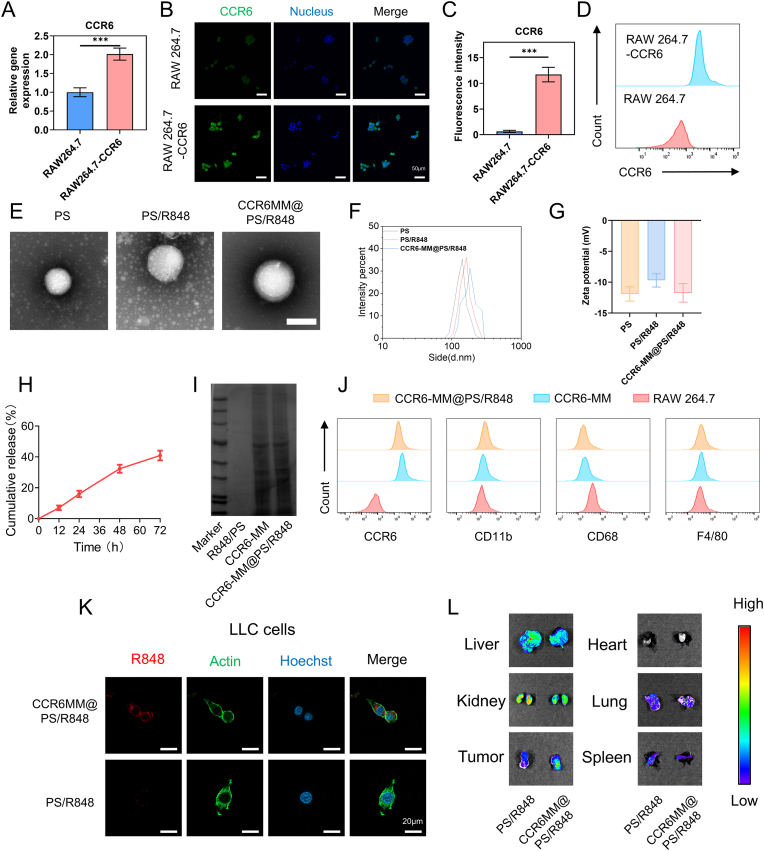
Characterization of CCR6-MM@PS/R848. A. q-PCR analysis of CCR6 mRNA expression in RAW264.7 cells (n = 3). (B) Immunofluorescence microscopy images depicting CCR6 expression in RAW264.7 cells. Scale bar = 100 μm. (C) Quantitative assessment of CCR6 expression derived from immunofluorescence images shown in panel B (n = 3). (D) Flow cytometry analysis of CCR6 surface expression on RAW264.7 cells. (E) Transmission electron microscopy (TEM) images of CCR6-MM@PS/R848 and its components. Scale bar = 100 nm. (F) Analysis of particle size for CCR6-MM@PS/R848 and its components. (G) Zeta potential measurements for CCR6-MM@PS/R848 and its components (n = 3). (H) Evaluation of R848 loading efficiency in CCR6-MM@PS/R848 (n = 3). (I) Coomassie Brilliant Blue staining of proteins in CCR6-MM@PS/R848 and its components. (J) Flow cytometry analysis of membrane protein expression (CCR6, CD11b, CD68, and F4/80) in CCR6-MM@PS/R848, CCR6-MM, and RAW264.7 cells. (K) Immunofluorescence images showing in vitro internalization of CCR6-MM@PS/R848. Scale bar = 100 μm. (L) In vivo targeting capability of CCR6-MM@PS/R848 assessed by fluorescence imaging. Data are presented as mean ± SD. Statistical significance was determined using one-way ANOVA with Bonferroni's multiple comparison test. ns: no significance, *P < 0.05, **P < 0.01, ***P < 0.001. (For interpretation of the references to colour in this figure legend, the reader is referred to the Web version of this article.)

we first established a RAW264.7 cell line overexpressing CCR6. The successful construction of this cell line was confirmed through multiple validation methods. Quantitative real-time PCR (q-PCR) analysis demonstrated a significant increase in CCR6 mRNA expression in the engineered RAW264.7 cells compared to the control group (Fig. 3A). Immunofluorescence staining revealed strong CCR6 protein expression on the cell surface, with distinct green fluorescence indicating the presence of CCR6 (Fig. 3B and C). Flow cytometry further confirmed the overexpression of CCR6 on the cell surface, with a marked shift in fluorescence intensity compared to control cells (Fig. 3D). These results collectively validated the successful establishment of the CCR6-overexpressing RAW264.7 cell line.

Subsequently, we extracted the cell membrane from the CCR6-overexpressing RAW264.7 cells to obtain CCR6-MM. This membrane was then used to prepare the CCR6-MM@PS/R848 complex. The PS liposome, PS/R848 liposome and CCR6-MM@PS/R848 hybrid liposomes were observed using TEM. The results indicated that all three types of nanovesicles maintained a vesicular structure. However, the introduction of the R848 drug led to an increase in the particle size of the PS/R848 liposome compared to the PS liposome. Additionally, the incorporation of the CCR6-MM cell membrane further significantly enlarged the particle size (Fig. 3E and F).

Furthermore, according to the calculations, the average particle sizes of PS, PS/R848, and CCR6-MM@PS/R848 were 139.20 ± 7.12 nm, 159.27 ± 6.45 nm, and 184.93 ± 12.22 nm, respectively, while their corresponding PDI values were 0.034 ± 0.006, 0.032 ± 0.008, and 0.056 ± 0.006 (Table S1). These results indicate that PS, PS/R848, and CCR6-MM@PS/R848 exhibit narrow particle size distributions and can be regarded as monodisperse systems. The zeta potentials of the PS liposome, PS/R848 liposome and CCR6-MM@PS/R848 hybrid liposomes were roughly similar (Fig. 3G). To evaluate the long-term stability of CCR6-MM@PS/R848 under simulated physiological conditions, CCR6-MM@PS/R848 was cultured in phosphate-buffered saline (PBS) and a medium containing 10 % fetal bovine serum (FBS) at 37 °C for 7 days. The particle size and Zeta potential of CCR6-MM@PS/R848 were measured at predetermined time points. As shown in Fig. S2 A–B, the average particle size and Zeta potential of CCR6-MM@PS/R848 in PBS remained largely unchanged, whereas in the medium containing 10 % FBS, the particle size exhibited a slight increase, while the Zeta potential remained stable. These results indicate that CCR6-MM@PS/R848 exhibits good stability under simulated physiological conditions. We evaluated the encapsulation efficiency and drug loading efficiency of R848 in CCR6-MM@PS/R848. The experimental results indicated that the encapsulation efficiency of CCR6-MM@PS/R848 was 8.32 %, while the drug loading efficiency was 53.35 %. Additionally, the cumulative release of R848 from CCR6-MM@PS/R848 over a period of 72 h was determined to be 43.2 % (Fig. 3H). After the preparation of CCR6-MM@PS/R848 hybrid liposomes, Confocal laser scanning microscopy was utilized to visualize the distribution of DiD-stained CCR6-MM and DiO-stained Lip, the red and green fluorescence signals from DiD and DiO, respectively, completely overlapped, resulting in a yellow fluorescence (Fig. S3). This indicated the successful fusion of the liposomal and CCR6-MM membranes within the hybrid nanoparticles. To characterize the protein composition of CCR6-MM@PS/R848, we employed Coomassie Brilliant Blue staining. Gel electrophoresis showed that CCR6-MM@PS/R848 and CCR6-MM exhibited similar protein profiles, indicating that the conjugation process did not significantly alter the protein composition (Fig. 3I). Flow cytometry analysis further revealed that the CCR6-MM@PS/R848 complex maintained the integrity of macrophage classical membrane receptors and exhibited high expression of CCR6 (Fig. 3J). These findings suggest that the CCR6-MM@PS/R848 complex retains the functional characteristics of the original cell membrane while incorporating the R848 component.

To validate the targeting capability of CCR6-MM@PS/R848, we utilized Cy5.5, a near-infrared fluorescent dye, to label R848. This allowed us to track the distribution and accumulation of the complex both at the cellular and in vivo levels. At the cellular level, we observed that CCR6-MM@PS/R848 exhibited significantly stronger red fluorescence in LLC cells compared to control groups, while its uptake by RAW264.7 macrophages was visibly enhanced—consistent with the inherent macrophage-homing property of macrophage-membrane-coated nanomedicines. Normal fibroblasts (L929) showed negligible fluorescence under all conditions, confirming minimal off-target entry into healthy stromal cells (Fig. 3K and Fig. S4). This selective tumor-cell and macrophage targeting indicates that the complex effectively delivers R848 to the tumor microenvironment while sparing normal fibroblasts. Mice bearing subcutaneous tumors were intravenously injected with Cy5.5-labeled CCR6-MM@PS/R848. Whole-body imaging revealed that CCR6-MM@PS/R848 produced visibly stronger fluorescence at the tumor site than PS/R848 group. Ex vivo imaging of excised organs showed prominent signals in liver, kidney and spleen across two groups; however, the CCR6-MM@PS/R848 group displayed markedly higher fluorescence at the tumor site. Semiquantitative analysis confirmed that the tumor-associated signal in the CCR6-MM@PS/R848 group was significantly greater than that in the PS/R848 group, underscoring the enhanced tumor-homing capacity conferred by the CCR6-engineered macrophage membrane (Fig. 3L and Figs. S5–S6). The results demonstrate that CCR6-MM@PS/R848 effectively targets CCR6-expressing cells and tissues, both in vitro and in vivo. The enhanced fluorescence observed in the CCR6-MM@PS/R848-treated groups suggests that the complex can efficiently deliver R848 to specific sites, potentially improving therapeutic efficacy while minimizing off-target effects. The accumulation in the liver and kidneys may reflect the natural clearance pathways of the body. Consistent with previous reports, these biomimetic nanocarriers are primarily cleared via the mononuclear phagocyte system and subsequently excreted by the liver and kidneys for degradation [37]. However, the pronounced signal observed at the tumor site suggests specific targeting capability. This targeting capability is crucial for developing targeted therapies that can enhance the immune response against cancer while reducing systemic toxicity.

### 3.4 Assessment of the biocompatibility of CCR6-MM@PS/R848

The primary objective of this chapter was to comprehensively evaluate the biocompatibility of CCR6-MM@PS/R848, a novel nanosponge formulation designed to target chemokine CCL20 in NSCLC. Understanding the biocompatibility of this formulation is crucial for its potential clinical application, as it ensures minimal cytotoxicity and adverse effects on normal cells and tissues.

**Fig. 4:**
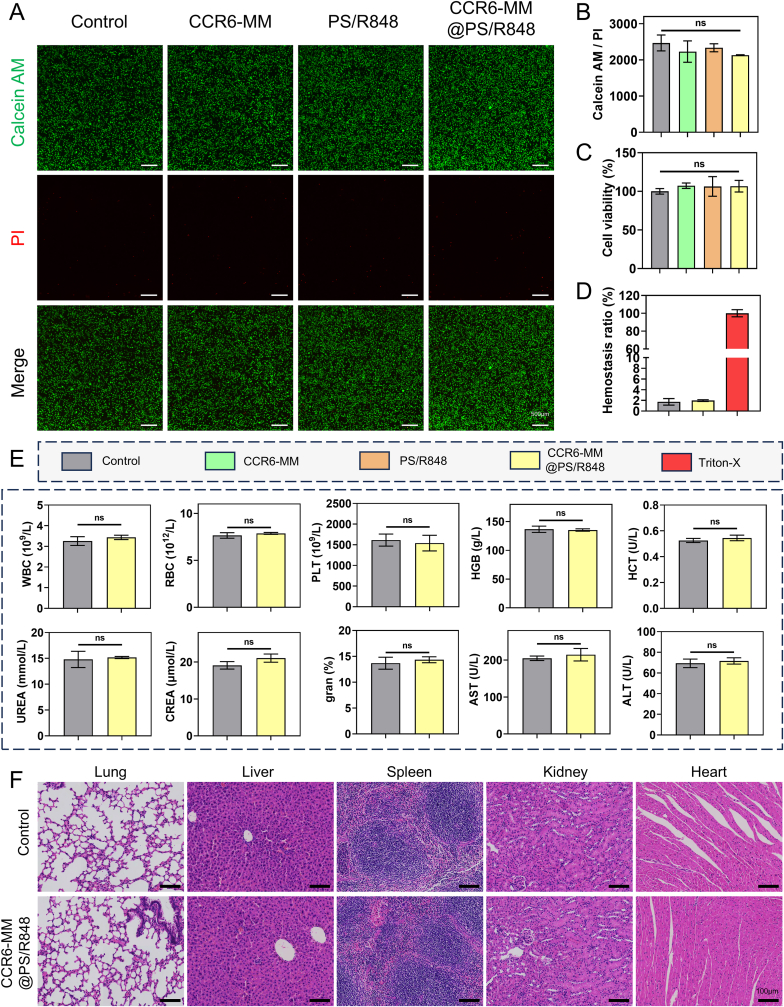
Biocompatibility Assessment of CCR6-MM@PS/R848. A. Live/dead staining of cells treated with CCR6-MM@PS/R848. Live cells are stained green with Calcein AM, while dead cells are stained red with propidium iodide (PI). Scale bar = 500 μm. B. Quantitative analysis of live/dead staining results from panel A (n = 3). C. Cell viability assessed by CCK8 assay after treatment with CCR6-MM@PS/R848 (n = 3). D. Quantitative results of hemolysis assay after treatment with CCR6-MM@PS/R848 (n = 3). E. Various blood indices in mice after treatment with CCR6-MM@PS/R848 (n = 3). F. Histological sections of major organs (heart, liver, spleen, lung, and kidney) stained with H&E after treatment with CCR6-MM@PS/R848. Scale bar = 100 μm. Data are presented as mean ± SD. Statistical significance was determined using one-way ANOVA with Bonferroni's multiple comparison test. ns: no significance, *P < 0.05, **P < 0.01, ***P < 0.001. (For interpretation of the references to colour in this figure legend, the reader is referred to the Web version of this article.)

Fig. 4A and B depict the results of the live-dead staining assay conducted on L929 cells treated with CCR6-MM@PS/R848. The absence of significant differences in cell viability among the various treatment groups suggests that CCR6-MM@PS/R848 does not induce cytotoxic effects on L929 cells. This finding is further corroborated by the CCK8 assay, which also indicate no significant differences in cell viability between the treatment groups (Fig. 4C). These in vitro results collectively demonstrate the excellent cellular biocompatibility of CCR6-MM@PS/R848, highlighting its potential for safe application in therapeutic settings.

The hemolysis assay results reveal that CCR6-MM@PS/R848 exhibits hemocompatibility similar to that of the negative control (Fig. 4D). This finding is critical, as it indicates that the nanosponge does not induce significant hemolysis, thereby reducing the risk of adverse reactions in the bloodstream. The preservation of red blood cell integrity is essential for maintaining normal physiological functions, and these results suggest that CCR6-MM@PS/R848 is unlikely to cause hemolytic complications.

The in vivo biocompatibility of CCR6-MM@PS/R848 was assessed through comprehensive hematological and biochemical analyses, as well as histopathological examination of major organs. The hematological and biochemical parameters, which show no significant differences between the CCR6-MM@PS/R848-treated group and the control group (Fig. 4E). This finding underscores the formulation's lack of systemic toxicity and its compatibility with the body's physiological environment. Furthermore, the histopathological sections of major organs (heart, liver, spleen, lung, and kidney) from the treated mice, revealing no signs of tissue damage or inflammation (Fig. 4F). The absence of organ toxicity is a key factor in the safe application of any therapeutic agent, and these results provide strong evidence of the biocompatibility of CCR6-MM@PS/R848 in vivo.

In summary, the results of this chapter demonstrate the high biocompatibility of CCR6-MM@PS/R848, both in vitro and in vivo. The lack of cytotoxicity, hemolysis, and organ toxicity associated with this formulation suggests that it is a promising candidate for further preclinical and clinical development.

### 3.5 Reprogramming of M2 macrophages to M1 phenotype by CCR6-MM@PS/R848

The TME is characterized by a high proportion of M2-type TAMs, which contribute to tumor progression, immune suppression, and resistance to therapy. M2-type macrophages promote tumor growth, angiogenesis, and metastasis, and are associated with poor prognosis in various cancers. Reprogramming M2-type macrophages to the M1 phenotype, which exhibits pro-inflammatory and anti-tumor properties, is a promising strategy to enhance the efficacy of cancer immunotherapy. In this study, we investigated the ability of CCR6-MM@PS/R848 to reprogram Raw264.7 macrophages from the M2 to the M1 phenotype.

**Fig. 5:**
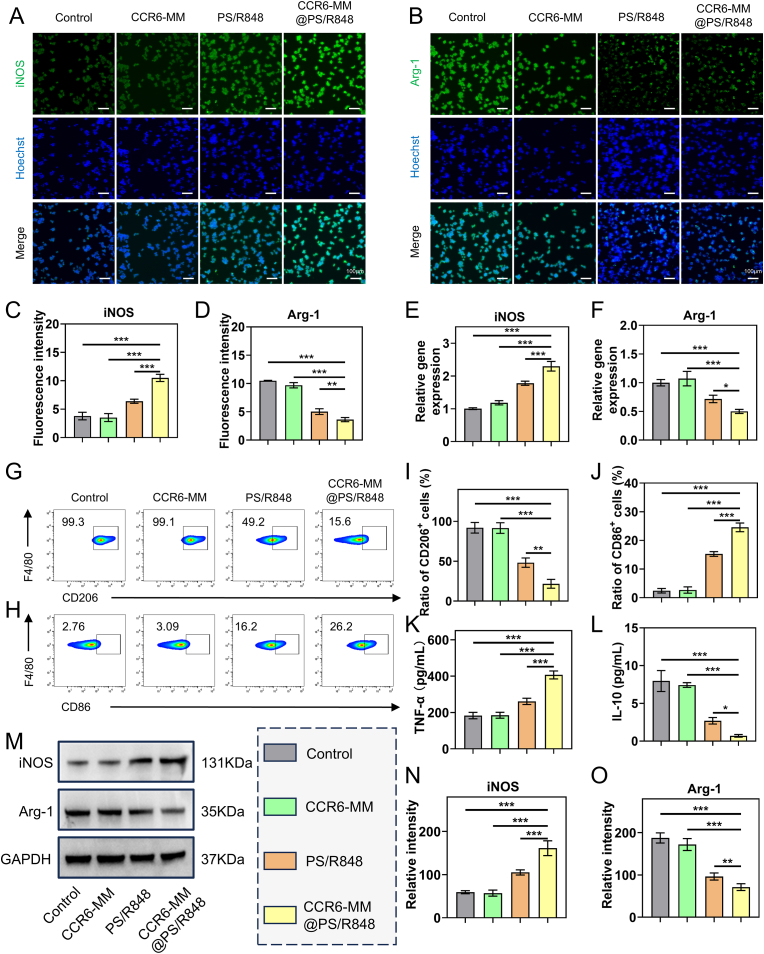
In Vitro Promotion of M1 Polarization in RAW264.7 Cells by CCR6-MM@PS/R848. A. Immunofluorescence images of RAW264.7 cells stained for iNOS (green) and nuclei (blue, Hoechst). Scale bar = 100 μm. B. Immunofluorescence images of RAW264.7 cells stained for Arg-1 (green) and nuclei (blue, Hoechst). Scale bar = 100 μm. C. Quantitative analysis of iNOS immunofluorescence intensity from panel A (n = 3). D. Quantitative analysis of Arg-1 immunofluorescence intensity from panel B (n = 3). E. qPCR analysis of iNOS mRNA expression in RAW264.7 cells (n = 3). F. qPCR analysis of Arg-1 mRNA expression in RAW264.7 cells (n = 3). G. Flow cytometry analysis of F4/80^+^CD206^+^ (M2 marker) RAW264.7 cells. H. Flow cytometry analysis of F4/80^+^CD86^+^ (M1 marker) RAW264.7 cells. I. Quantitative analysis of F4/80^+^CD206^+^ cells from panel G (n = 3). J. Quantitative analysis of F4/80^+^CD86^+^ cells from panel H (n = 3). K. ELISA analysis of TNF-α levels in the supernatant of RAW264.7 cells (n = 3). L. ELISA analysis of IL-10 levels in the supernatant of RAW264.7 cells (n = 3). M. Western blot analysis of iNOS and Arg-1 protein expression in RAW264.7 cells. N. Quantitative analysis of iNOS protein expression from panel M (n = 3). O. Quantitative analysis of Arg-1 protein expression from panel M (n = 3). Data are presented as mean ± SD. Statistical significance was determined using one-way ANOVA with Bonferroni's multiple comparison test. ns: no significance, *P < 0.05, **P < 0.01, ***P < 0.001. (For interpretation of the references to colour in this figure legend, the reader is referred to the Web version of this article.)

To simulate the TME, Raw264.7 cells were polarized to the M2 phenotype using IL-4 as a control group. High-magnification bright-field microscopy revealed that M2 macrophages exhibited an elongated spindle morphology; in contrast, cells treated with CCR6-MM@PS/R848 adopted a polygonal shape, consistent with an M1 phenotype (Fig. S7).The results of immunofluorescence experiments, which demonstrate that CCR6-MM@PS/R848 significantly enhanced the fluorescence intensity of iNOS, a marker of M1 macrophages, while reducing the fluorescence intensity of Arg-1, a marker of M2 macrophages, compared to other groups (Fig. 5A–D). This indicates that CCR6-MM@PS/R848 has a strong capacity to reprogram M2 macrophages to the M1 phenotype. The results of q-PCR experiments, which further validate the immunofluorescence findings by showing similar trends in the expression levels of iNOS and Arg-1 (Fig. 5E–F). These results collectively suggest that CCR6-MM@PS/R848 effectively induces the M1 phenotype in Raw264.7 macrophages. The results of flow cytometry analysis, which demonstrate that CCR6-MM@PS/R848 significantly increased the proportion of F4/80 and CD86 double-positive cells, indicative of M1 macrophages, while decreasing the proportion of F4/80 and CD206 double-positive cells, indicative of M2 macrophages (Fig. 5G–J). These findings reinforce the notion that CCR6-MM@PS/R848 promotes the reprogramming of M2 macrophages to the M1 phenotype. ELISA results showed a similar trend: the M1-macrophage signature cytokine TNF-α was increased, whereas the M2-macrophage signature cytokine IL-10 was decreased (Fig. 5K–L). The results of Western blot experiments, which confirm the reprogramming effect of CCR6-MM@PS/R848 at the protein level (Fig. 5M–O). The expression of iNOS was significantly upregulated in the CCR6-MM@PS/R848 group, while the expression of Arg-1 was downregulated. This further supports the conclusion that CCR6-MM@PS/R848 effectively reprograms M2 macrophages to the M1 phenotype. In summary, the results of this chapter demonstrate that CCR6-MM@PS/R848 has a robust capacity to reprogram M2 macrophages to the M1 phenotype, as evidenced by multiple experimental approaches. This reprogramming ability is crucial for enhancing the anti-tumor immune response and overcoming immune suppression in the TME.

### 3.6 In vivo inhibition of lung tumor growth by CCR6-MM@PS/R848

**Fig. 6:**
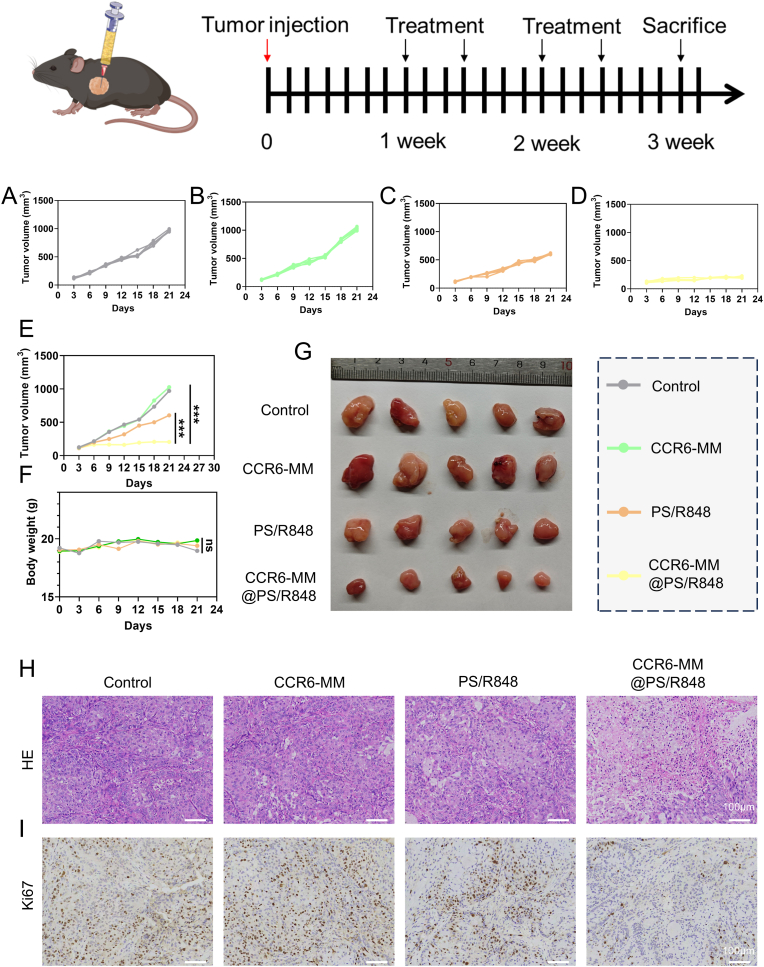
In Vivo Antitumor Efficacy of CCR6-MM@PS/R848. A-E. Tumor growth curves of mice in different treatment groups (n = 5). Tumor volume was measured every 3 days. F. Changes in body weight of mice in different treatment groups (n = 5). G. Representative images of tumors from mice in different treatment groups (n = 5). H. H&E staining of tumor sections from different treatment groups. Scale bar = 100 μm. I. Immunofluorescence staining of tumor sections from different treatment groups. Scale bar = 100 μm.

The primary objective of this chapter was to evaluate the in vivo efficacy of CCR6-MM@PS/R848 in inhibiting the growth of subcutaneous LLC tumors in a mouse model. The tumor growth curves of the different treatment groups (Fig. 6A–E). The results show that the CCR6-MM@PS/R848 group exhibited significant inhibition of tumor growth compared to other groups. This finding suggests that CCR6-MM@PS/R848 has a potent antitumor effect in the LLC mouse model. Meanwhile, the changes in mouse body weight across the different treatment groups (Fig. 6F). The absence of significant differences in body weight among the groups indicates that CCR6-MM@PS/R848 treatment did not cause substantial systemic toxicity. This result further supports the biocompatibility of CCR6-MM@PS/R848, suggesting that it can be safely administered in vivo. The physical appearance of tumors excised at the end of the observation period (Fig. 6G). The tumors from the CCR6-MM@PS/R848 group were the smallest, visually demonstrating the inhibitory effect of the treatment on tumor growth. Histological assessment of the tumors revealed that the CCR6-MM@PS/R848 group exhibited significant areas of necrosis (Fig. 6H). Additionally, Ki67 staining showed the lowest positivity rate in the CCR6-MM@PS/R848 group, indicating that cell proliferation was markedly suppressed. These histological findings collectively highlight the efficacy of CCR6-MM@PS/R848 in inhibiting tumor growth and inducing tumor necrosis.

In summary, the results of this chapter demonstrate that CCR6-MM@PS/R848 effectively inhibits the growth of subcutaneous LLC tumors in mice while maintaining good biocompatibility. The significant reduction in tumor size, along with the histological evidence of tumor necrosis and reduced cell proliferation, underscores the therapeutic potential of CCR6-MM@PS/R848.

### 3.7 In vivo reprogramming of macrophages from M2 to M1 phenotype by CCR6-MM@PS/R848

**Fig. 7:**
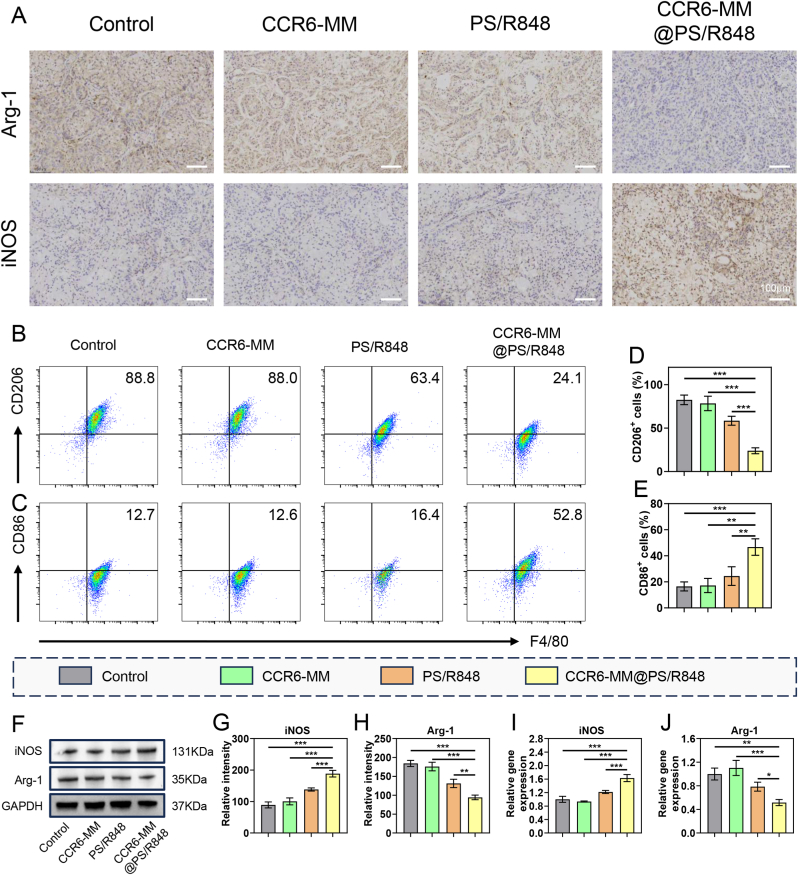
In Vivo Promotion of M1 Polarization by CCR6-MM@PS/R848. A. Immunohistochemical staining for Arg-1 and iNOS in tumor tissues from different treatment groups. Scale bar = 100 μm. B. Flow cytometry analysis of F4/80^+^CD206^+^ (M2 marker) cells in tumor tissues from different treatment groups. Scale bar = 100 μm. C. Flow cytometry analysis of F4/80^+^CD86^+^ (M1 marker) cells in tumor tissues from different treatment groups. Scale bar = 100 μm. D. Quantitative analysis of F4/80^+^CD206^+^ cells from panel B (n = 3). E. Quantitative analysis of F4/80^+^CD86^+^ cells from panel C (n = 3). F. Western blot analysis of iNOS and Arg-1 protein expression in tumor tissues from different treatment groups. G. Quantitative analysis of iNOS protein expression from panel F (n = 3). H. Quantitative analysis of Arg-1 protein expression from panel F (n = 3). I. qPCR analysis of iNOS mRNA expression in tumor tissues from different treatment groups (n = 3). J. qPCR analysis of Arg-1 mRNA expression in tumor tissues from different treatment groups (n = 3). Data are presented as mean ± SD. Statistical significance was determined using one-way ANOVA with Bonferroni's multiple comparison test. ns: no significance, *P < 0.05, **P < 0.01, ***P < 0.001.

The primary objective of this chapter was to evaluate the in vivo efficacy of CCR6-MM@PS/R848 in reprogramming TAMs from the M2 phenotype to the M1 phenotype within the TME. The immunohistochemistry (IHC) results, which show that the CCR6-MM@PS/R848 group exhibited a significant reduction in Arg-1 positivity and a marked increase in iNOS positivity (Fig. 7A). These findings indicate a robust reprogramming effect of CCR6-MM@PS/R848 on macrophages, shifting them from the M2 to the M1 phenotype. This shift is crucial for enhancing the anti-tumor immune response, as M1 macrophages are known to promote inflammation and inhibit tumor growth. The results of flow cytometry analysis, which further validate the reprogramming effect of CCR6-MM@PS/R848 (Fig. 7B–E). The proportion of F4/80 and CD206 double-positive cells, indicative of M2 macrophages, was significantly reduced in the CCR6-MM@PS/R848 group. Conversely, the proportion of F4/80 and CD86 double-positive cells, indicative of M1 macrophages, was significantly increased. These findings collectively demonstrate the ability of CCR6-MM@PS/R848 to reprogram TAMs from the M2 to the M1 phenotype in vivo. The results of Western blot analysis performed on macrophages sorted by flow cytometry (Fig. 7F–H). The CCR6-MM@PS/R848 group exhibited the highest expression of iNOS protein and the lowest expression of Arg-1 protein. These results further support the reprogramming effect of CCR6-MM@PS/R848 at the protein level, confirming its ability to induce a pro-inflammatory M1 phenotype in macrophages. The q-PCR results, which validate the reprogramming effect of CCR6-MM@PS/R848 at the mRNA level (Fig. 7I–J). The expression levels of iNOS and Arg-1 were consistent with the protein expression results, further supporting the reprogramming capacity of CCR6-MM@PS/R848.

In summary, the results of this chapter demonstrate that CCR6-MM@PS/R848 effectively reprograms TAMs from the M2 to the M1 phenotype in vivo, as evidenced by multiple experimental approaches. This reprogramming ability is crucial for enhancing the anti-tumor immune response and overcoming immune suppression in the TME.

### 3.8 In vitro inhibition of lung cancer cell migration and invasion by CCR6-MM@PS/R848

**Fig. 8:**
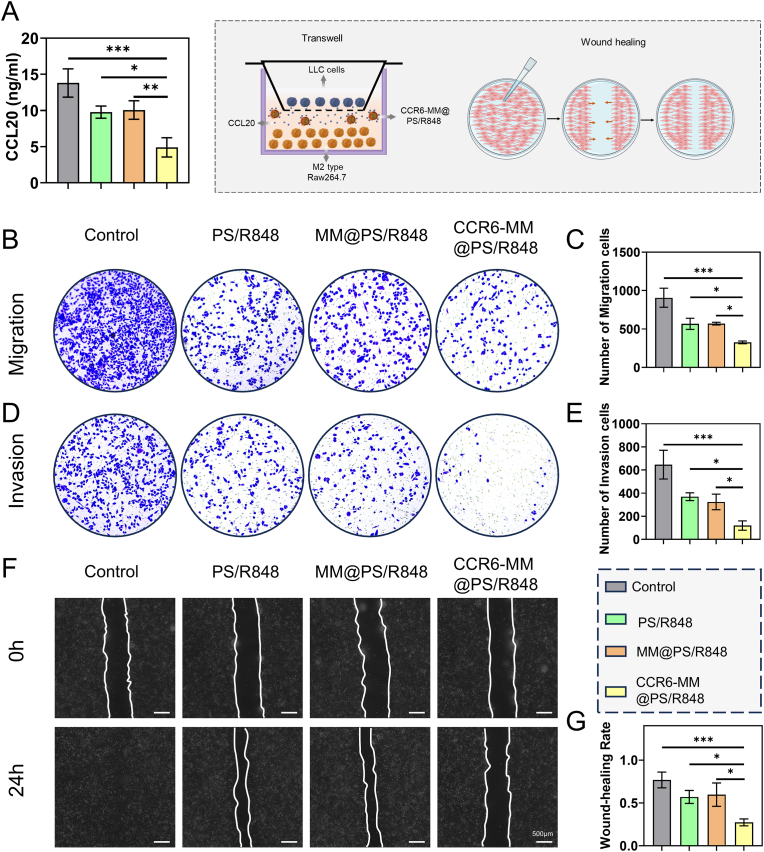
In Vitro Suppression of LLC Cell Migration and Invasion by CCR6-MM@PS/R848-Co-cultured RAW264.7 Cell Supernatants. A. ELISA analysis of CCL20 levels in the supernatant of RAW264.7 cells from different treatment groups (n = 3). B. Transwell migration assay of LLC cells treated with supernatants from different RAW264.7 cell treatment groups. C. Quantitative analysis of LLC cell migration from panel B (n = 3). D. Transwell invasion assay of LLC cells treated with supernatants from different RAW264.7 cell treatment groups. E. Quantitative analysis of LLC cell invasion from panel D (n = 3). F. Scratch assay of LLC cells treated with supernatants from different RAW264.7 cell treatment groups. G. Quantitative analysis of LLC cell migration from panel F (n = 3). Data are presented as mean ± SD. Statistical significance was determined using one-way ANOVA with Bonferroni's multiple comparison test. ns: no significance, *P < 0.05, **P < 0.01, ***P < 0.001.

This chapter aimed to determine whether CCR6-MM@PS/R848 could reduce the production of CCL20 and inhibiting the migratory and invasive capabilities of LLC cells. As shown in Fig. 8A, CCR6-MM@PS/R848 markedly reduced CCL20 levels in macrophage-conditioned medium compared with all control groups, an effect attributable to both (i) R848-driven M2→M1 repolarization and (ii) CCR6-mediated adsorption of residual CCL20. This reduction in CCL20 levels is crucial, as CCL20 is known to promote the migration and invasion of cancer cells. The results of the Transwell migration and invasion assays (Fig. 8B–E). These experiments demonstrate that the supernatant from CCR6-MM@PS/R848-treated macrophages significantly reduced the migratory and invasive capabilities of LLC cells. This finding indicates that the reprogramming of macrophages by CCR6-MM@PS/R848 effectively inhibits the metastatic potential of LLC cells. Figures F–G show the results of the scratch assay, which further supports the inhibitory effect of CCR6-MM@PS/R848 on LLC cell migration. The scratch assay results indicate that the supernatant from CCR6-MM@PS/R848-treated macrophages significantly reduced the closure of the scratch wound, suggesting a marked reduction in cell migration.

In summary, the results of this chapter demonstrate that CCR6-MM@PS/R848 effectively reduces the production of CCL20 and thereby inhibiting the migration and invasion of LLC cells. This ability is crucial for inhibiting tumor metastasis in the TME.

### 3.9 In vivo inhibition of lung cancer metastasis by CCR6-MM@PS/R848

**Fig. 9:**
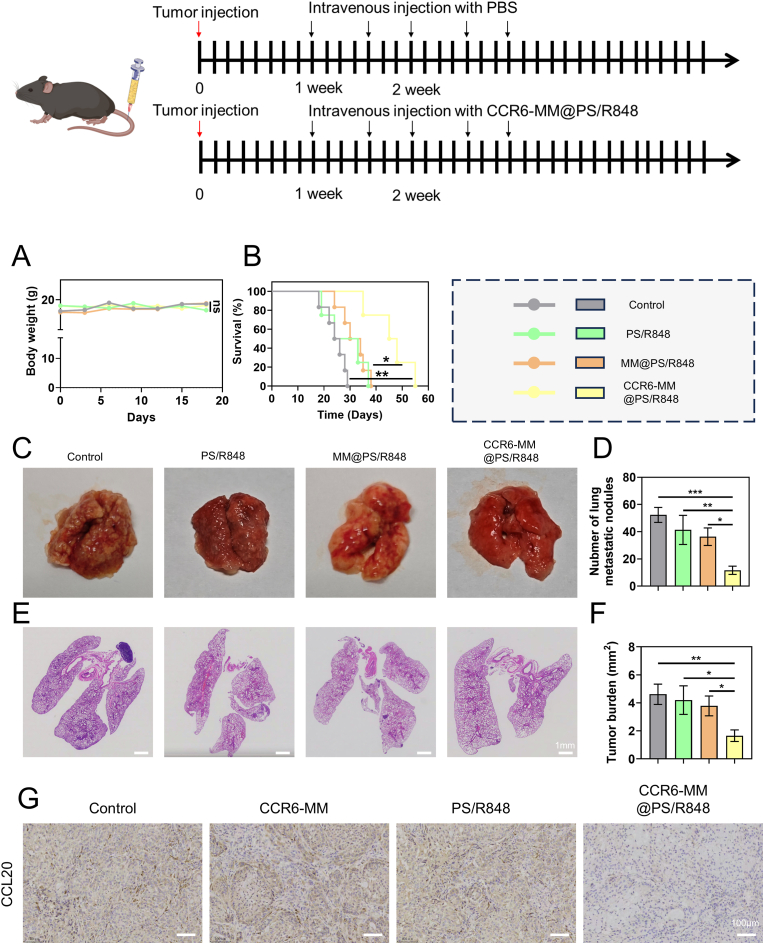
In Vivo Antitumor Metastasis by CCR6-MM@PS/R848. A. Changes in body weight of mice in different treatment groups (n = 5). B. Kaplan-Meier survival curves of mice in different treatment groups (n = 5). C. Representative images of lung metastasis in mice from different treatment groups (n = 5). D. Quantitative analysis of the number of lung metastatic nodules from panel C (n = 5). E. H&E staining of lung sections from different treatment groups. Scale bar = 100 μm. F. Quantitative analysis of lung metastasis severity based on H&E staining from panel E (n = 5). G. Immunohistochemical staining for CCL20 in tumor tissues from different treatment groups. Scale bar = 100 μm. Data are presented as mean ± SD. Statistical significance was determined using one-way ANOVA with Bonferroni's multiple comparison test, except for survival analysis, which was performed using the log-rank test. ns: no significance, *P < 0.05, **P < 0.01, ***P < 0.001.

To evaluate the therapeutic potential of CCR6-MM@PS/R848 against metastatic NSCLC, we first determined whether LLC represents a high-CCL20 tumor model. Comparative ELISA of five murine tumor lines (MC38, CT26, 4T1, B16-F10, Panc02) revealed that LLC secreted the highest amount of CCL20 (Fig. S8), validating its use as a high-CCL20 model. Then, we employed an experimental lung metastasis model generated by tail-vein injection of LLC-Luc cells (1 × 10^6^ cells/mouse) into 6- to 8-week-old female C57BL/6 mice. Treatments were initiated seven days after tumor inoculation, mice were randomly assigned to four groups (n = 10) and received intravenous injections of saline, PS/R848, MM@PS/R848, or CCR6-MM@PS/R848 twice per week for a total of four doses. Body weight was recorded every other day as a surrogate for systemic toxicity, and survival was monitored until humane endpoints. At day 28, a subset of animals (n = 5) was euthanized for enumeration of surface metastatic nodules, histopathology, and immunohistochemical analysis of CCL20 expression. The changes in mouse body weight across different treatment groups (Fig. 9A). The absence of significant differences in body weight indicates that CCR6-MM@PS/R848 treatment did not cause substantial systemic toxicity, further supporting its biocompatibility. The survival curves of the mice, demonstrating that the CCR6-MM@PS/R848 group exhibited significantly prolonged survival compared to other groups (Fig. 9B). This finding suggests that CCR6-MM@PS/R848 effectively inhibits tumor progression and improves survival outcomes. The physical appearance of the lungs and the number of metastatic nodules in each treatment group (Fig. 9C and D). The CCR6-MM@PS/R848 group showed a marked reduction in the number of metastatic nodules, visually demonstrating the inhibitory effect of the treatment on lung metastasis. Histological examination of the lungs further supports these findings (Fig. 9D–F). H&E staining revealed fewer metastatic lesions in the lungs of mice treated with CCR6-MM@PS/R848 compared to other groups. The IHC results, which show a significant reduction in CCL20-positive rate in the CCR6-MM@PS/R848 group (Fig. 9G). The ELISA results also show a significant reduction in CCL20 in the CCR6-MM@PS/R848 group (Fig. S9). To further verify that CCL20 derived from tumor cells drives metastasis, we generated LLC-CCL20-KO cells. In a parallel experiment, mice receiving LLC-CCL20-KO cells exhibited a marked reduction in pulmonary nodules relative to parental LLC controls (Fig. S10), confirming that tumor-derived CCL20 is functionally important for metastatic progression. This finding indicates that CCR6-MM@PS/R848 effectively reduces the production of CCL20, a chemokine known to promote cancer cell migration and invasion. The reduction in CCL20-positive rate is consistent with the observed decrease in metastatic potential, suggesting that CCR6-MM@PS/R848 exerts its anti-metastatic effects by modulating the TME.

In summary, the results of this chapter demonstrate that CCR6-MM@PS/R848 effectively inhibits lung cancer metastasis in a mouse model while maintaining good biocompatibility. The significant reduction in metastatic nodules and CCL20-positive rate, along with prolonged survival, underscores the therapeutic potential of CCR6-MM@PS/R848.

## 4 Conclusion

CCR6-MM@PS/R848 represents the first bifunctional nanoplatform that unites active CCL20 sequestration with in-situ immune activation within a single construct. By membrane-displaying the CCR6 receptor, the system achieves tumor-selective CCL20 scavenging and simultaneously repolarizes immunosuppressive M2-like macrophages toward an M1 phenotype through encapsulated R848. This dual "target-and-neutralize" strategy converts a chemokine-rich, immune-evasive microenvironment into an immune-permissive milieu, thereby suppressing metastatic progression without eliciting systemic toxicity.

Beyond non-small-cell lung cancer, the modular architecture can be readily adapted to other malignancies driven by distinct chemokine axes, offering a versatile blueprint for precision immuno-metabolic therapy. The membrane-cloaked design not only prolongs circulation time and enhances tumor accumulation via active recognition, but also provides a generic strategy for co-delivery of immune modulators and targeting ligands. By integrating receptor-mediated ligand capture with localized immune reprogramming, this work establishes a new paradigm for synergistic, precision cancer immunotherapy that can be customized to diverse oncologic indications.

## CRediT authorship contribution statement

**Libao Liu:** Writing – review & editing, Writing – original draft, Validation, Software, Methodology, Data curation, Conceptualization. **Yonghui Wu:** Writing – review & editing, Writing – original draft, Validation, Data curation, Conceptualization. **Weibin Wu:** Writing – review & editing, Writing – original draft, Validation, Funding acquisition, Data curation, Conceptualization. **Zining Liu:** Validation, Software, Methodology, Formal analysis. **Bolin Chen:** Validation, Software, Formal analysis. **Guanghong Wu:** Software, Project administration, Methodology. **Zhe Ji:** Validation, Software, Methodology, Formal analysis, Data curation. **Jiannan Xu:** Writing – review & editing, Supervision, Project administration, Data curation. **Shuai Huang:** Writing – review & editing, Supervision, Data curation, Conceptualization. **Kai Zhang:** Writing – review & editing, Supervision, Data curation, Conceptualization.

## Ethics approval and consent to participate

Animal experimental protocols were approved by the Animal Ethics Committee of Shenzhen lingfutuopu co., Ltd. (TOPGM-LACUC-2023-0221, Shenzhen, China).

## Declaration of competing interest

The authors declare that they have no known competing financial interests or personal relationships that could have appeared to influence the work reported in this paper.

## Data Availability

Data will be made available on request.
