# Supplementary material for: Targeting chemokine-driven metastasis in non-small cell lung cancer: Development and evaluation of chemokine nanosponges for therapy
Source: Mater Today Bio. 2025 Nov 4;35:102511. doi: 10.1016/j.mtbio.2025.102511 (PMC12859670; doi:10.1016/j.mtbio.2025.102511)
Supplement: Multimedia component 1 [file mmc1.docx]

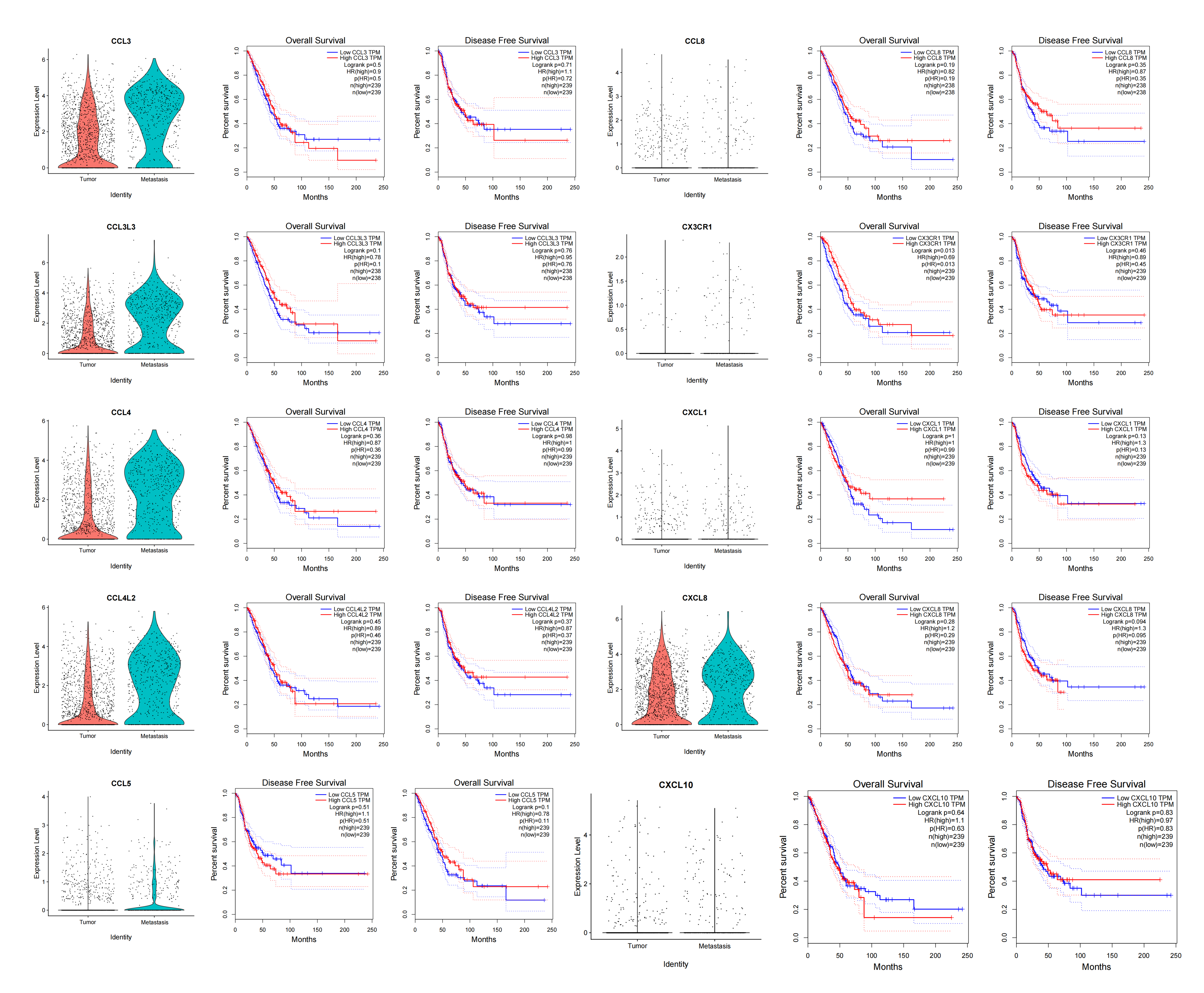


Figure. S1. Expression levels and prognostic analysis of genes in the cytokine–cytokine receptor pathway across tumor and metastasis cohorts.


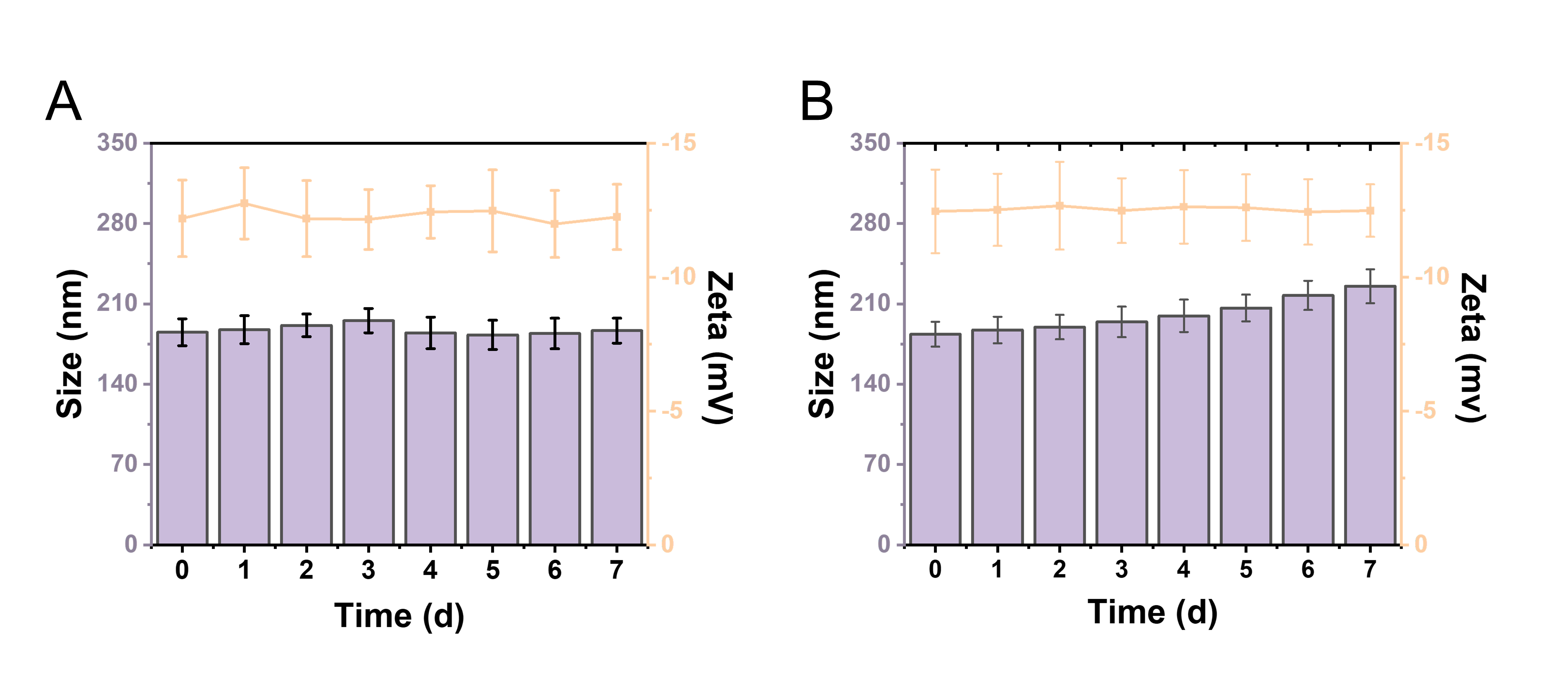


Figure S2. The average particle size and Zeta potential changes of CCR6-MM@PS/R848 in (A)PBS and (B) medium containing 10%FBS.


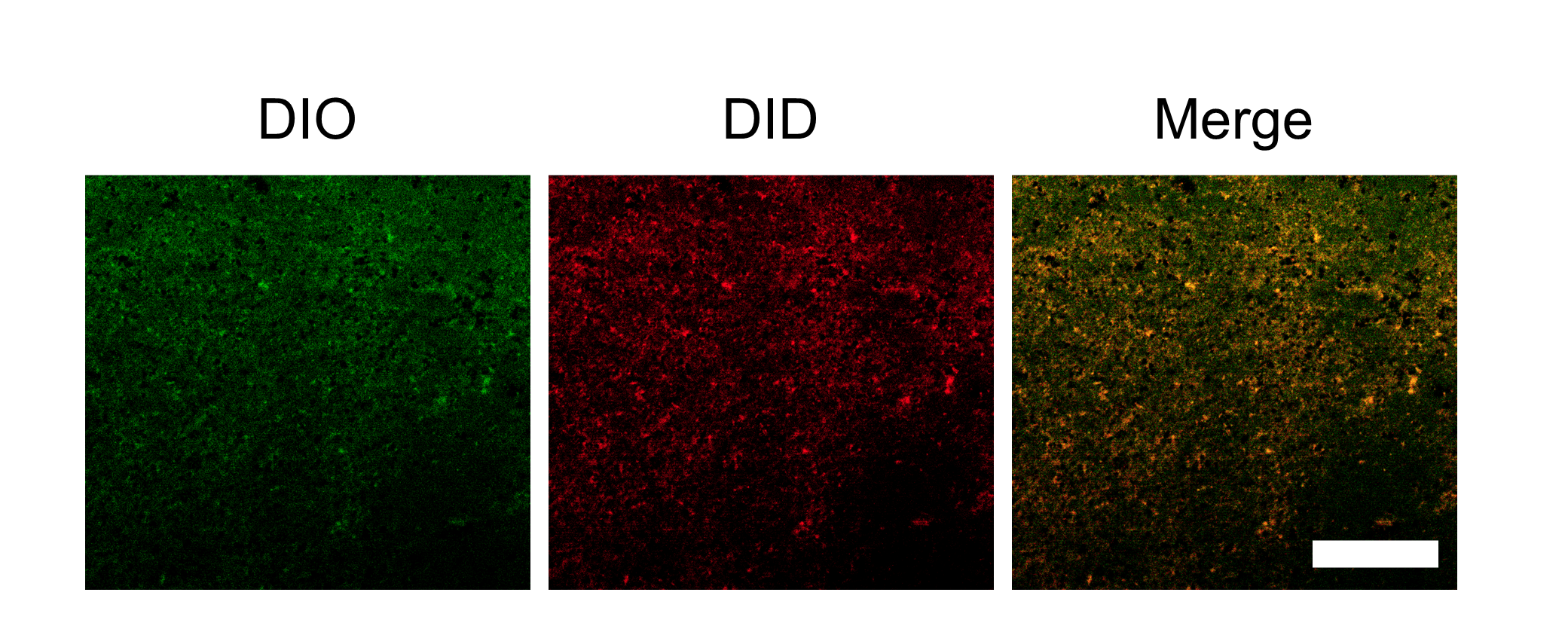


Figure S3. DID and DIO staining for CCR6-MM@PS/R848.


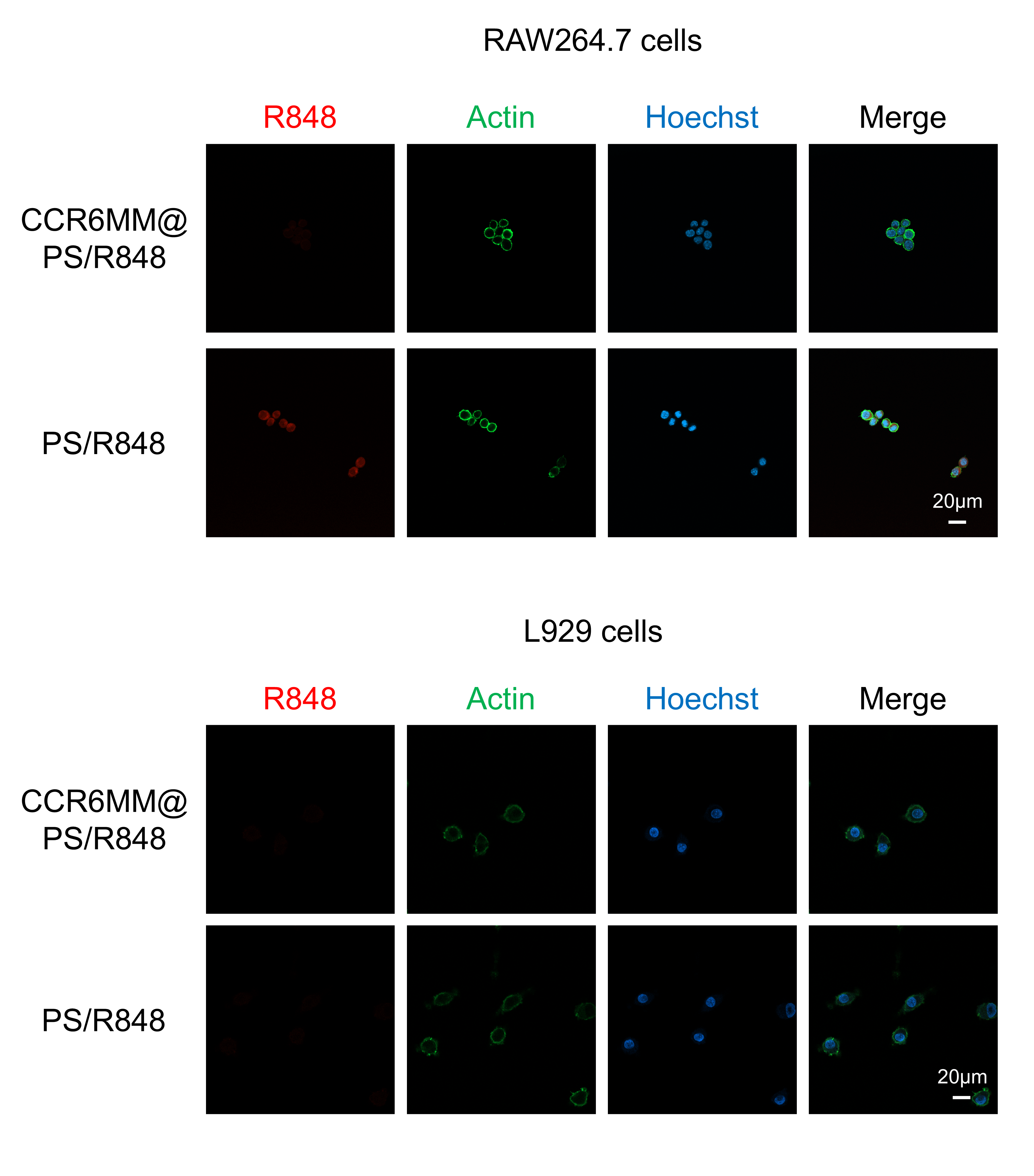


Figure. S4. Immunofluorescence images of in vitro internalization of CCR6-MM@PS/R848 in RAW264.7 and L929 cells. Scale bar = 20 µm.


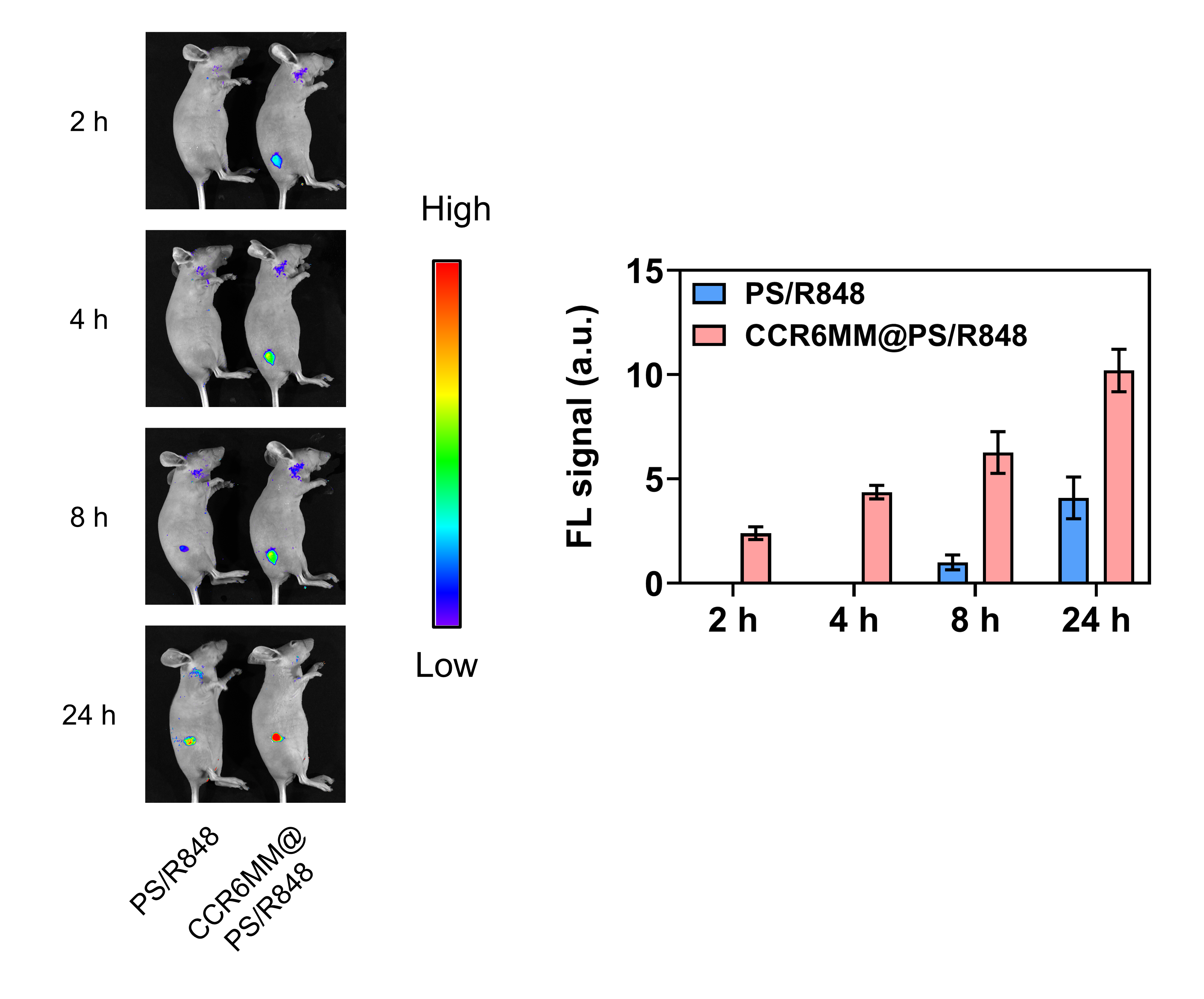


Figure S5. In vivo imaging of CCR6MM@PS/R848 tumor targeting (n = 3). *P < 0.05, **P < 0.01, ***P < 0.001.


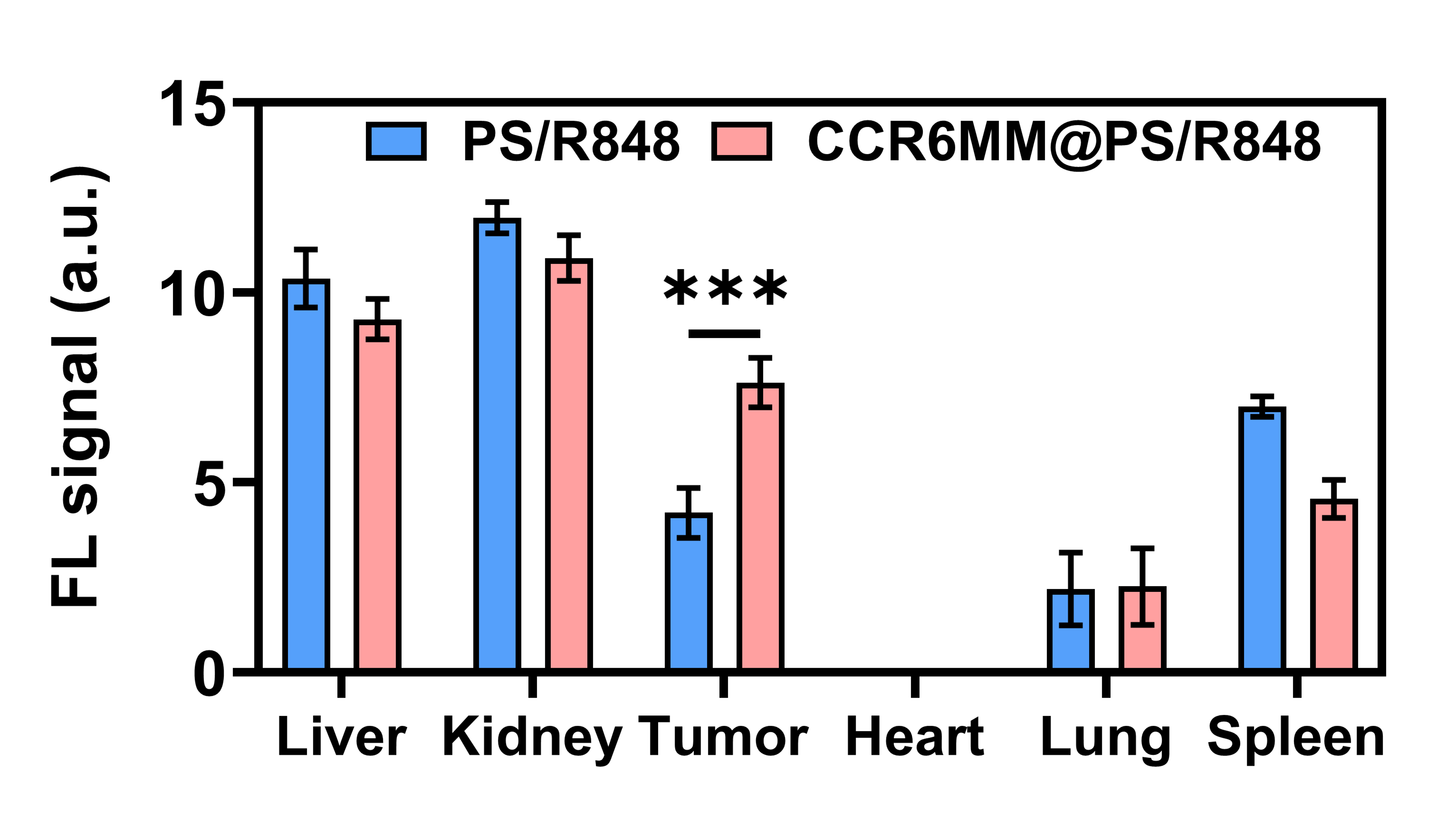


Figure S6. Quantitative analysis of the in vivo tumor-targeting capability of CCR6-MM@PS/R848 as determined by fluorescence imaging (n = 3). *P < 0.05, **P < 0.01, ***P < 0.001.


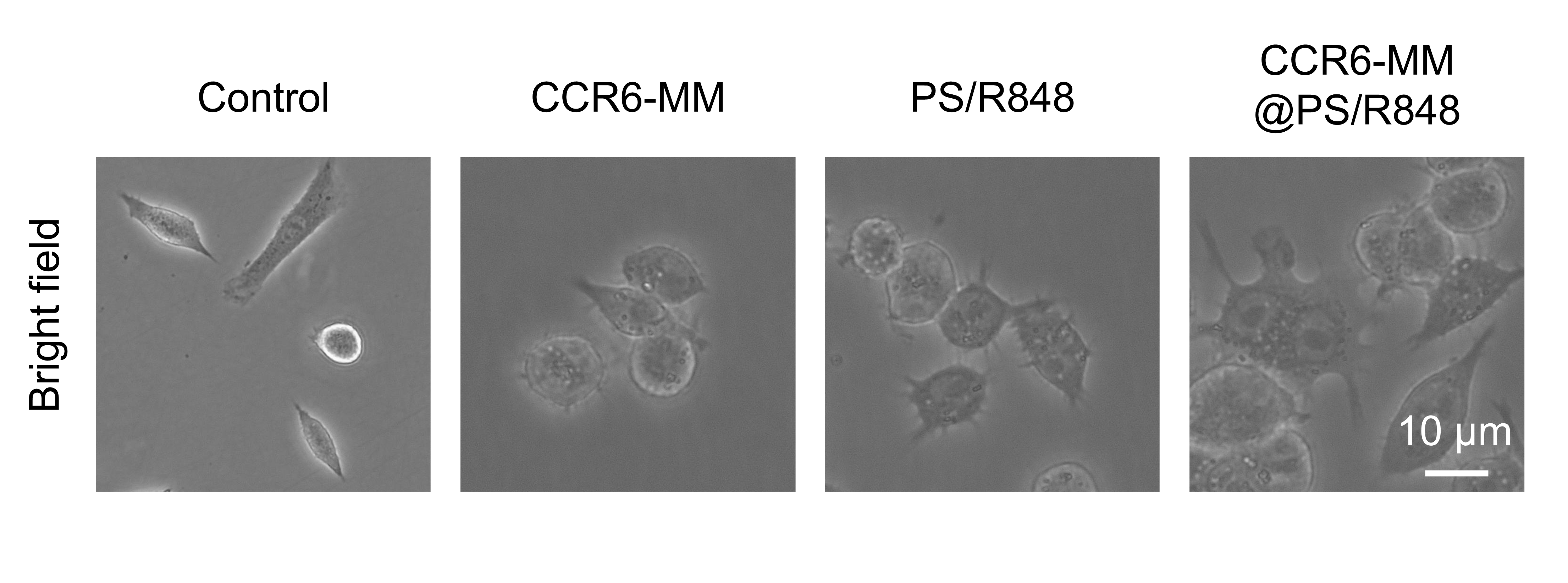


Figure S7. High-magnification bright-field images of RAW264.7 cells in each treatment group.


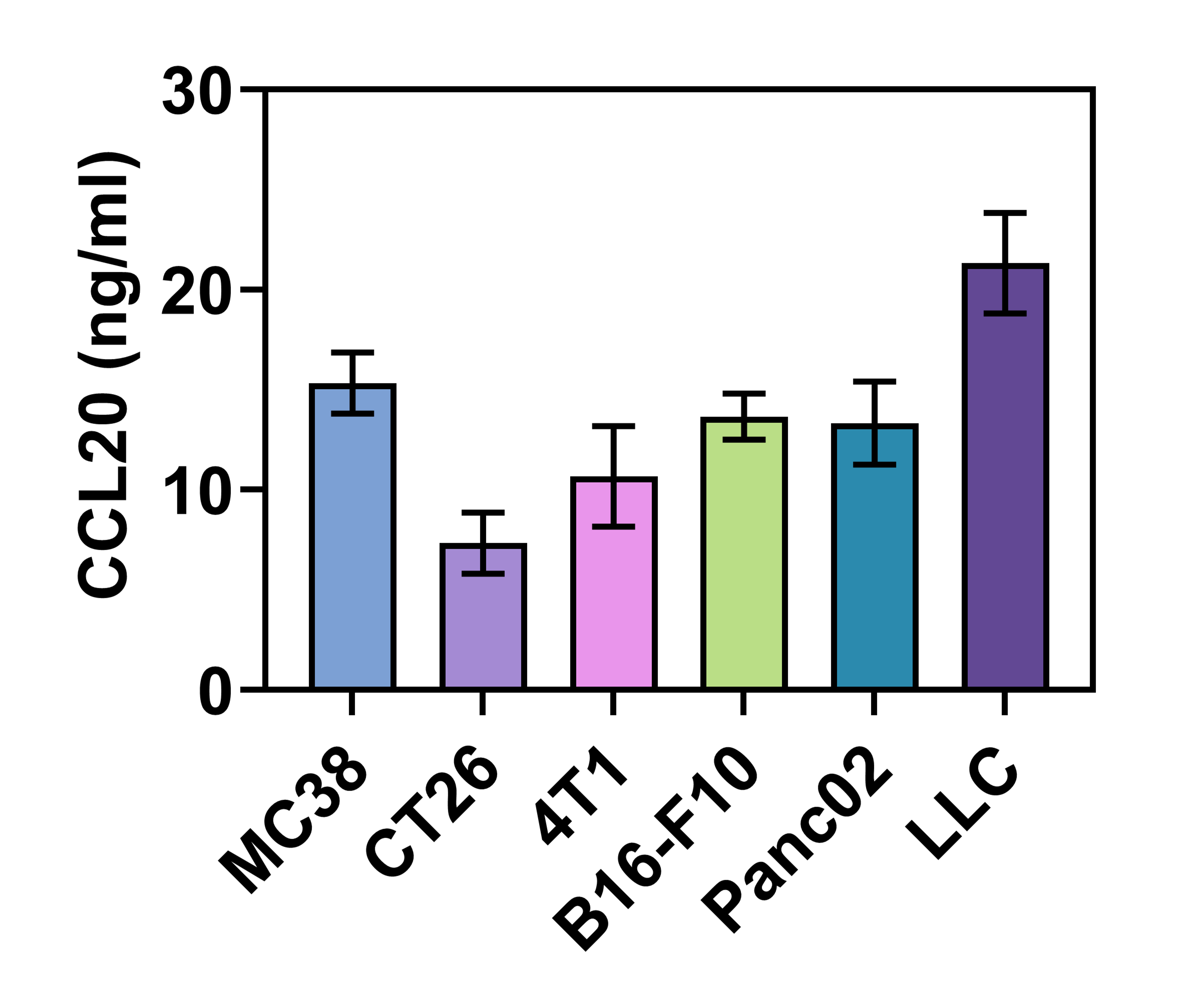


Figure S8. ELISA quantification of CCL20 secretion across multiple tumor cell lines (n = 3). *P < 0.05, **P < 0.01, ***P < 0.001.


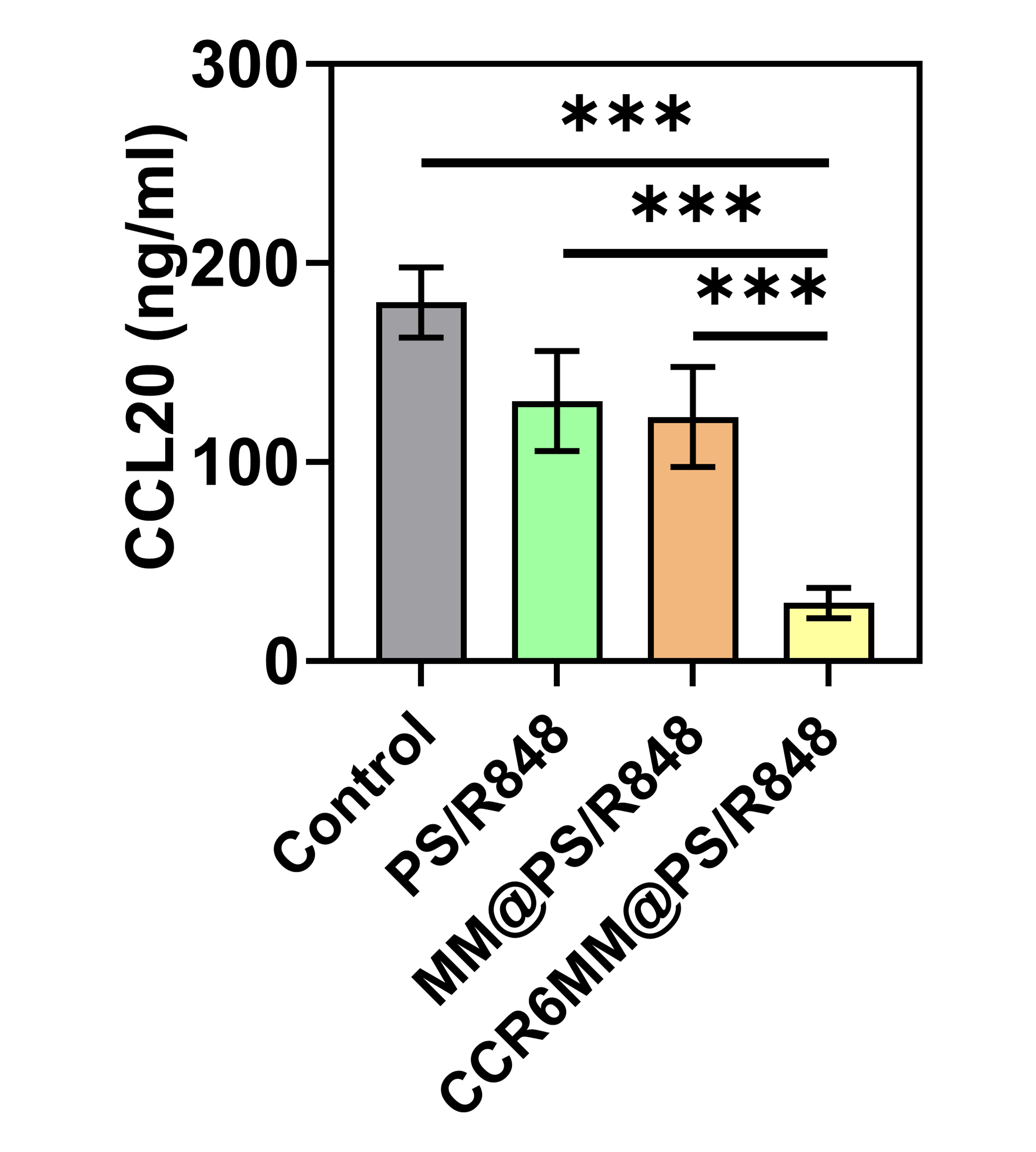


Figure S9. ELISA analysis of CCL20 levels in tumor (n = 3). *P < 0.05, **P < 0.01, ***P < 0.001.


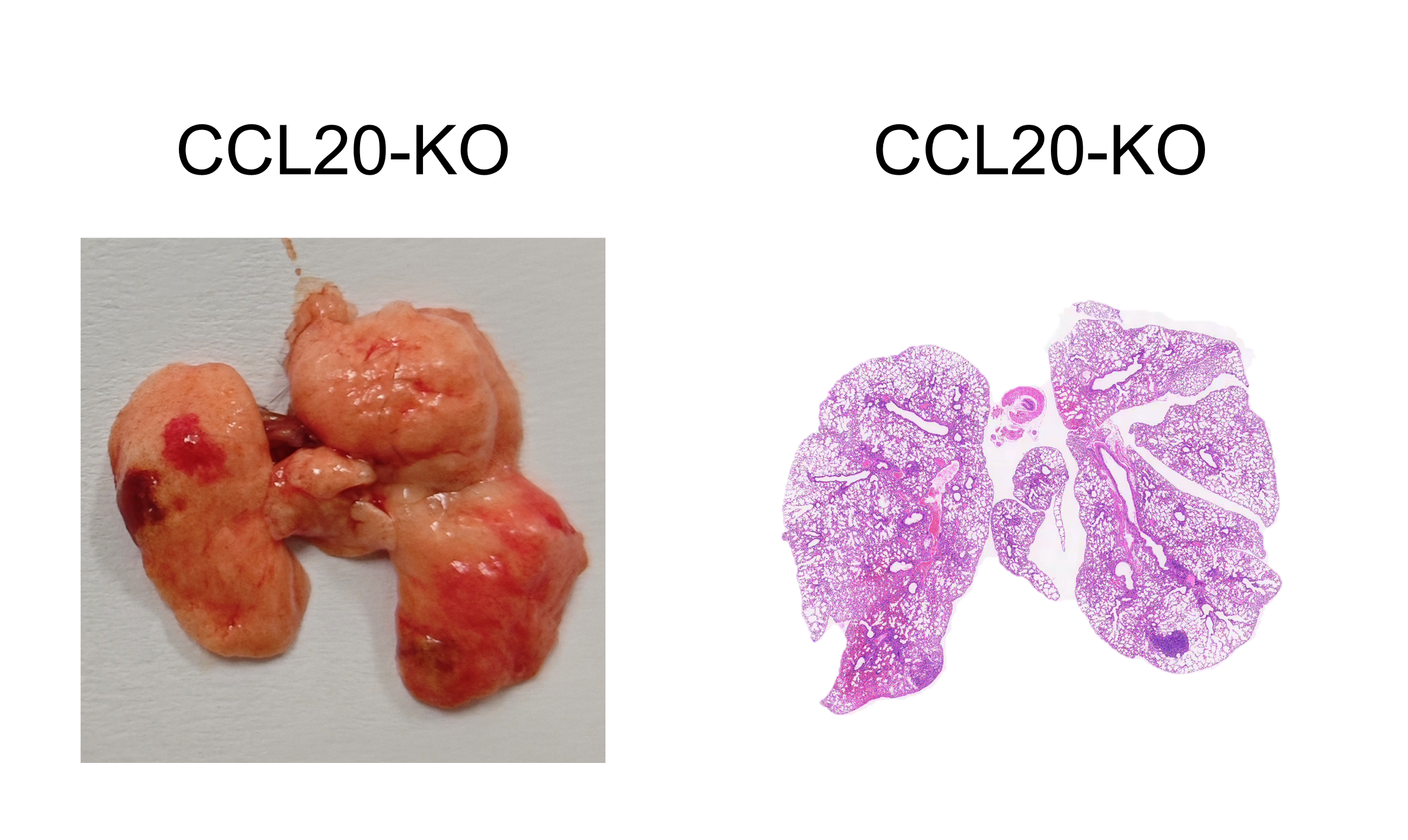


Figure S10. Representative images of lung metastasis in CCL20-KO mice.

Table S1. Size and PDI of PS, PS/R848, CCR-6-MM@PS/R848

| **Parameters** | **Size (nm)** | **PDI** |
| --- | --- | --- |
| PS | 139.20±7.12 | 0.034±0.006 |
| PS/R848 | 159.27±6.45 | 0.032±0.008 |
| CCR-6-MM@PS/R848 | 184.93±12.22 | 0.056±0.006 |
